# Adult organotypic brain slice cultures recapitulate extracellular matrix remodeling in hemorrhagic stroke

**DOI:** 10.3389/fncel.2025.1722240

**Published:** 2026-01-27

**Authors:** Benjamin J. Hewitt, Lauren Roberts, James A. Roberts, Daniel Fulton, Lisa J. Hill, Philip Kitchen, Roslyn M. Bill, Hannah F. Botfield

**Affiliations:** 1 Department of Biomedical Sciences, School of Infection, Inflammation and Immunology, College of Medicine and Health, University of Birmingham, Birmingham, United Kingdom; 2 Aston Institute for Membrane Excellence, Aston University, Birmingham, United Kingdom; 3 Department of Inflammation and Ageing, School of Infection, Inflammation and Immunology, College of Medicine and Health, University of Birmingham, Birmingham, United Kingdom; 4 Birmingham Centre for Neurogenetics, University of Birmingham, Birmingham, United Kingdom

**Keywords:** extracellular matrix, hemorrhage, hemorrhagic stroke, organotypic 3D culture, slice culture, organotypic brain slice culture, adult brain slices

## Abstract

Haemorrhagic stroke is a devastating condition characterized by vessel rupture and free blood within the brain parenchyma or cerebrospinal fluid (CSF) filled spaces. Across the major subtypes of hemorrhagic stroke (subarachnoid, intracerebral, and intraventricular hemorrhages), the presence of blood in the CSF generates significant tissue damage in the first 72 h after the event, known as early brain injury (EBI). EBI includes neuroinflammation, blood-brain barrier breakdown and dysregulation of extracellular matrix (ECM) dynamics. ECM dysfunction has been shown to trigger fibrosis of the cortical blood vessels, limiting normal CSF circulation and resulting in the buildup of metabolic waste or the development of post-hemorrhagic hydrocephalus. Limiting or preventing this fibrosis may therefore reduce the rate of morbidity experienced by survivors, providing a potential avenue for non-surgical treatment to reduce secondary brain injury post-stroke. Despite this, current *in vivo* approaches fail to differentiate between the effect of blood products and secondary consequences including intracranial pressure (ICP) elevation and mass effect. Here, we describe an adult rat organotypic brain slice culture (OBSC) model of hemorrhagic stroke which enables the identification of the effect of blood products on ECM dysregulation. We demonstrate the distribution of key cell types across a time course of 0, 3 and 7 days in culture, indicating that such cultures are viable for a minimum of 7 days. Using immunofluorescence staining, Western blotting and RNA sequencing, we show that exposure of OBSCs to lysed blood markedly increases ECM deposition around cortical blood vessels. This is accompanied by dysregulation of ECM regulatory genes and upregulation of inflammation and oxidative stress-related genes, successfully recapitulating the changes seen in human stroke survivors. This versatile *ex vivo* model provides a translational platform to further understanding of hemorrhagic stroke pathophysiology and develop or trial novel therapeutics prior to progression to *in vivo* stroke studies.

## Graphical Abstract

**Figure:**
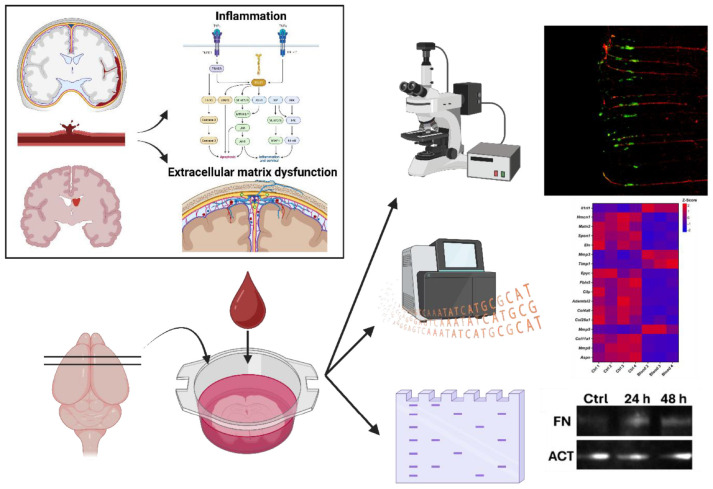
Overview of the hypothesis and experimental workflow used to investigate extracellular matrix (ECM) remodelling in hemorrhagic stroke. Blood was added to adult organotypic brain slice cultures (OBSCs), followed by analysis of ECM and inflammation using tissue staining, Western blotting and RNA sequencing. Created in https://BioRender.com.

## 1 Introduction

Hemorrhagic stroke is a devastating condition characterized by vessel rupture and free blood within the brain parenchyma or cerebrospinal fluid (CSF)-filled spaces, and may be split into intracerebral hemorrhage (ICH), subarachnoid hemorrhage (SAH) and intraventricular hemorrhage (IVH) depending on the location of the bleed. While hemorrhagic stroke only accounts for approximately 20% of stroke cases (Donkor, 2018), the risk of mortality or profound disability is markedly greater than that posed by ischemic stroke (Waziry et al., 2020).

SAH is most commonly triggered by a ruptured aneurysm causing blood to leak into the CSF in the subarachnoid space, with blood also entering the ventricles in higher grade SAH. Early brain injury (EBI) is the injury that occurs in the brain within 72 h of an SAH and develops due to mechanical factors and the direct effect of free blood. SAH causes an increase in intracranial pressure (ICP), decreased cerebral blood flow (resulting in global ischemia), blood brain barrier (BBB) breakdown, brain edema, oxidative stress and neuroinflammation, which ultimately leads to neurodegeneration (Lauzier et al., 2023).

Extracellular matrix (ECM) dynamics in and around the brain are also affected following SAH. There is a fine balance between ECM deposition and degradation which is particularly evident within blood vessel architecture. Under pathological conditions, BBB dysfunction is associated with the degradation of ECM in the basement membrane, mainly through matrix metalloproteinases. This can then release growth factors such as the pleiotropic cytokine transforming growth factor-β1 (TGF-β1) which in turn stimulates vascular smooth muscle cells (SMCs) to promote ECM deposition and repair the BBB (Hu et al., 2023). SAH can result in excessive or dysregulated ECM deposition around major blood vessels on the surface of the brain, contributing to the development of cerebral vasospasm and subsequent delayed cerebral ischemia (Hu et al., 2023). Furthermore, elevated TGF-β1 levels have been implicated in the development of subarachnoid fibrosis and ECM dysfunction in CSF drainage pathways after SAH which can lead to the development of post-hemorrhagic hydrocephalus (Motohashi et al., 1995; Douglas et al., 2009; Chen et al., 2017; Hewitt et al., 2025). Understanding the pathways and mechanisms involved in ECM dysfunction may provide therapeutic targets for reducing further brain injury following SAH.

In both human patients and animal models of SAH, it can be difficult to differentiate between the response of the brain to the mechanical factors that follow a stroke (ICP elevation, mass effect) and the direct reaction of brain tissue to blood products, which is important to understand the pathogenesis of common sequalae to SAH and focus the development of future therapeutics to treat these. In contrast, *ex vivo* models allow us to differentiate between these factors to identify and further understand disease processes. Organotypic brain slices cultures (OBSCs), which were first described in the 1950s for the study of membrane potentials in the cerebral cortex (Li and McIlwain, 1957), have been used in the study of neurodegenerative diseases (Croft et al., 2019), invasive glioma (Eisemann et al., 2018) and epilepsy (Magalhães et al., 2018) amongst many other uses. OBSCs provide a three-dimensional tissue architecture and are widely considered to be more representative of the *in vivo* state than cell cultures due to the presence of neurons, glial cells and endothelial cells in a native tissue architecture (Humpel, 2015). OBSCs are typically prepared from neonatal brain tissues slices as these tend to be more resistant to the mechanical trauma caused by sectioning, making them easier to culture (Alaylioğlu et al., 2020). However, these neonatal models are unlikely to recapitulate the complexity or maturity of adult tissues, as is shown by different responses to sectioning. For this reason, adult OBSC models are essential in modeling a disease which primarily affects middle to older-aged adults (Jacobs et al., 2002). Whilst adult rodent OBSCs are now well established, there has been very limited prior exploration of their application to hemorrhagic stroke research. Attempts at short-term (minutes to hours) hemorrhagic stroke brain slice models have been reported with a focus on neuronal survival, though it is not clear whether they were from adult or neonatal animals (Mokrushin et al., 2008). Therefore, the development and validation of an adult OBSC system capable of modeling hemorrhagic stroke remains an important challenge.

Here, we describe the generation and validation of OBSCs derived from healthy adult rats and their application to the *ex vivo* modeling of post-hemorrhagic stroke ECM dysfunction and inflammation. *Ex vivo* recapitulation of ECM dynamics may provide an opportunity for furthering understanding of the molecular mechanisms underlying this key factor in post-stroke brain injury and offer a platform in which to trial novel therapeutics prior to use *in vivo*.

## 2 Materials and methods

### 2.1 Tissue culture

All tissue culture was performed under aseptic conditions using a class II microbiological safety cabinet (Monmouth Guardian T1200). Tissues were cultured at 37°C, 5% CO_2_, and 100% relative humidity.

Dissection media for OBSC generation comprised Earle’s balanced salt solution supplemented to a final concentration of 25 mM HEPES. OBSC culture media comprised 1:1 Dulbecco’s Modified Eagle Media (DMEM):Neurobasal A, supplemented with final concentrations of 2% (v/v) B-27, 2 mM L-glutamine and 1% penicillin/streptomycin solution.

### 2.2 Experimental animals

Male Sprague-Dawley rats were group housed with access to food and water *ad libitum*, on a 12-h light/dark cycle. All studies were conducted at the University of Birmingham, and both the establishment and investigators held relevant UK Home Office licenses. Animals were monitored daily and group housed, with no regulated procedures performed. Animals were euthanized according to Schedule 1 of the Animals (Scientific Procedures) Act 1986 at a weight of 130–150 g by intraperitoneal injection of 200 mg/mL pentobarbitol, 4 mL/kg and decapitated following cessation of circulation. Blood was captured in a sterile, un-heparinized 50 mL centrifuge tube and stored at −20°C until required. No a priori sample size calculations were performed due to the exploratory nature of this work, instead opting for a minimum sample size of *N* = 3.

### 2.3 Organotypic brain slice culture generation

**FIGURE 1:**
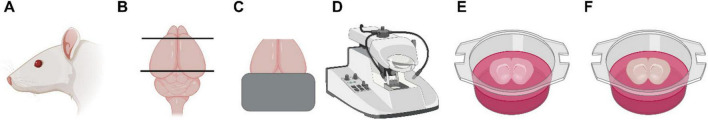
Steps in the generation of adult rat brain OBSCs. **(A)** Sacrifice and brain removal. **(B)** Removal of olfactory bulbs and cerebellum, including production of a flat edge on the caudal aspect of the cerebrum. **(C)** Mounting of brain (caudal face down) onto vibratome chuck with cyanoacrylate glue. **(D)** Vibratome slicing of brain (250 μM sections, 80 Hz, 1.25 mm amplitude). **(E)** Brain slices added to 6-well cell culture inserts. **(F)** Media removed to bring brain slice to air-liquid interface. Created in https://BioRender.com.

The brain was rapidly removed from the skull and the cerebellum and olfactory bulbs excised with a sterile scalpel. A flat face was produced on the rostral and caudal edges of the brain by cutting (Figure 1), and the brain adhered to the chuck of a vibratome (7000smz, Campden Instruments) (caudal side down) with cyanoacrylate glue. The brain was then mounted into the vibratome bath, containing 250 mL dissection medium on ice, bubbled with medical oxygen at 2 psi. 250 μM sections were produced with the vibratome set to a 1.25 mm amplitude at 80 Hz and sections stored in the dissection media bath until completion.

Slices were then transferred to 6-well polyethylene culture inserts (657641, Greiner) using a fine paintbrush and spatula. To facilitate this, 1 ml of culture medium was initially added to each insert to enable floating of the slice, which was removed once the slice was correctly positioned. Culture inserts were added to a standard 6-well plate (3516, Corning), and 1.5 mL of OBSC culture medium added to the bottom well with the top insert containing the brain slice left dry. Slices were then either fixed immediately or on day *in-vitro* (DIV) 3 or 7 using 4% paraformaldehyde (PFA) in PBS for 1 h at room temperature, followed by two, 1-h phosphate buffered saline (PBS) washes at room temperature prior to storage in PBS at 4°C.

### 2.4 Blood-treated OBSCs

OBSCs destined for blood treatment as a model of hemorrhagic stroke were generated as described above and exposed to blood immediately or after 7 days of pre-culture.

Blood from all animals was pooled, vortexed and shaken vigorously to break up clots, and diluted 1:1 with PBS. 6, 24, or 48 h prior to the experiment endpoint, 175 μL of diluted blood (or PBS control) was added to the wells assigned for blood treatment after removal of an equal volume of medium. Brain slices for immunohistochemical analysis were then fixed as previously described, prior to washing through two changes of PBS and storage at 4°C.

Slices destined for protein or RNA extraction were dissected under a surgical microscope, with the cortex removed (taking care to avoid capturing any tissue from the corpus callosum), snap frozen in liquid nitrogen and stored at −80°C.

### 2.5 Immunohistochemical staining and imaging

OBSCs were divided into two hemispheres prior to staining. A full protocol for staining and clearing of OSBCs can be found in Supplemental material (“Staining and clearing of OBSCs”). Antibodies were diluted in PBS with 0.2% (v/v) triton X-100, 6% (v/v) goat serum and 20% (v/v) DMSO, with 0.2 mL of diluted antibody (Table 1) added to each slice in a 24-well plate. Imaging was performed using an AxioScan Z.1 (Zeiss) for blood exposed sections and an AxioScan 7 (Zeiss) for timepoint studies maintaining the same imaging settings across all slides within an analysis group.

**TABLE 1:** Antibody dilutions used in immunofluorescent staining of OBSCs.

Antibody	Dilution
Anti-rat endothelial cell antigen 1 (RECA-1), mouse	1:100
Anti-fibronectin, rabbit	1:400
Anti-alpha smooth muscle actin (αSMA), mouse	1:500
Anti-cleaved caspase 3 (CCP3), rabbit	1:500
Anti-NeuN, mouse	1:500
Anti-GFAP, mouse	1:400
Anti-collagen IV, rabbit	1:500
Anti-IBA1, rabbit	1:500
AF488 conjugated anti-rabbit IgG, highly cross-absorbed	1:1,000
AF594 conjugated anti-mouse IgG, highly cross-absorbed	1:1,000

### 2.6 Image processing and analysis

Image processing was performed using FIJI (Schindelin et al., 2012), with the operator blinded to the treatment or culture conditions of each image by displaying only numerical filenames. Representative images shown have been enhanced for display by correcting uneven illumination using the BaSiC plugin for FIJI (Peng et al., 2017), followed by background subtraction (250 px radius) and modification of image window and level, performed uniformly across all images within a comparison group or stain. No image enhancement was performed on images prior to image analysis.

Mean pixel intensity per unit area was calculated by maximum intensity projection, user selection of 5 (NeuN, GFAP, IBA1, αSMA, and RECA-1) or 3 (CCP3, two slices to give 6 total) ROIs per image, followed by background subtraction (50 pixel radius) in FIJI, automated measurement of the ROI pixel intensity and division of this value by the area of the ROI.

Fibronectin and collagen IV quantification was performed using a FIJI macro made available both on GitHub^1^ and in Supplementary material. In brief, this performed a maximum intensity projection and presented the user with an image in the red channel only (corresponding to blood vessel staining with RECA-1). Five regions of interest (ROIs) were then manually selected in the cortex of each slice and processed for background subtraction (100 pixel radius for RECA-1 channel, 1,000 pixel radius for fibronectin and collagen IV), followed by automatic thresholding of blood vessels (RenyiEntropy method) and ECM proteins (Triangle method), with image calculation then performed to find the average of these two sets of features.

### 2.7 Protein extraction and Western blotting

Protein extraction was performed by mechanical homogenization in 250 μL radioimmunoprecipitation assay (RIPA) buffer, supplemented with protease and phosphatase inhibitors per manufacturers guidance. Samples were then incubated on a roller at 4°C for 2 h and centrifuged at 10,000 × *g* for 20 min at 4°C. Supernatant was then harvested, aliquoted and stored at −80°C until required. Protein concentration was assessed by BCA assay, following the manufacturer’s instructions. SDS-PAGE was performed using 2.5 μg of protein in a 20 μL volume, containing 5 μL LDS sample buffer and 2 μL reducing agent, on 4–12% bis-tris polyacrylamide gels using MOPS-SDS running buffer. Electrophoresis was performed at 120 V for 90 min. Proteins were then transferred to a nitrocellulose membrane using NuPAGE transfer buffer at 25 V for 1 h. Blocking was performed by immersion in 5% (w/v) skimmed milk in tris-buffered saline with 0.1% (v/v) Tween-20 (TBST) for 1 h at room temperature. Blots were then incubated in primary antibodies for fibronectin or collagen IV and alpha-actin diluted in blocking buffer for 1 h at room temperature, washed three times in TBST (15 min each, room temperature), and incubated in secondary antibody (1:5,000 anti-IgG HRP conjugated) for 1 h at room temperature. Blots were then washed 3x in TBST (10 min each, room temperature) prior to immersion in chemiluminescent substrate for 5 min (room temperature) and imaging (G:Box chemi XRQ, Syngene).

### 2.8 RNA extraction, sequencing, and RT-qPCR

Samples were mechanically disrupted and homogenized in 1 mL TRIzol and 200 μL chloroform was then added. Samples were then mixed by shaking and centrifuged at 12,000*g for 15 min at 4°C. The upper aqueous phase was removed and transferred to a new tube, and an equal volume of molecular grade 70% ethanol added. RNA was then extracted from the sample with an RNEasy mini extraction kit (Qiagen), following the manufacturer’s instructions for a 40 μL elution volume in RNase free water. RNA was then quantified by spectrophotometery (N50 NanoPhotometer, Implen). Sample quality control (integrity, purity and concentration via Agilent 5400) and paired-end mRNA sequencing was performed by a contracted 3rd party (Novogene, United Kingdom) using a NovaSeq X Plus (Illumina) at a read depth of 20 million reads per sample.

Alignment against a Sprague Dawley rat genome (rn_celera) was performed by Novogene, followed by mapping to the same reference genome using the featureCounts tool (Liao et al., 2014) on the Galaxy platform (Abueg et al., 2024), followed by quality control using MultiQC (Ewels et al., 2016). Analysis of differentially expressed genes was performed with DESeq2 (Love et al., 2014), with comparisons made of expression in blood-treated samples against an untreated control. Differentially expressed genes were defined as those with an adjusted *p-*value (*pAdj)* < 0.05, and a fold change cutoff of 2 was used for display of DEGs.

Gene ontology (GO) analysis was performed on all differentially expressed genes (with no fold-change cutoff) using goseq (Young et al., 2010), with gene categories retrieved from the rn6 assembly due to unavailability of gene categories for the rn_celera assembly. All visualization of RNA-sequencing data was performed using GraphPad Prism 10.3. Over-representation analysis (ORA) was performed using the Kyoto encyclopedia of Genes and Genomes (KEGG) methodology (Kanehisa et al., 2025) using WebGestalt (Elizarraras et al., 2024). Protein-protein interaction (PPI) analysis was performed using Cytoscape 3.10 with the STRING plugin (Szklarczyk et al., 2023). ECM gene ORA was performed through comparison to the Matrisome ECM gene set (Naba et al., 2012) using the R package clusterProfiler (Yu et al., 2012).

Validation of the top six most upregulated and top six most downregulated genes was performed using RT-qPCR. In brief, cDNA conversion was performed with a Tetro cDNA kit using Oligo(dT)18 primers and 100 ng of RNA. cDNA was then further diluted by addition of 20 μL molecular biology grade water. RT-qPCR mastermix comprised 100 μL SYBR green, 78 μL water and 1 μL of each forward and reverse primer (reconstituted to 100 μM). A total of 9 μL mastermix was added to each well of a 384-well PCR plate, followed by 1 μL cDNA. RT-qPCR was performed using a QuantStudio 5, with reaction conditions shown in Supplementary Table 1. Oligonucleotide primers used are shown in Supplementary Table 2.

### 2.9 Statistical analysis

All statistical analysis was performed in GraphPad Prism 10.3 unless otherwise stated, with a significance level of 0.05. Statistical tests are described in the relevant figure legends. All data were tested for normality using Shapiro-Wilk tests (Supplementary material, “Statistical analysis—normality testing”).

RT-qPCR data were analyzed with QBase+ (Biogazelle, Belgium) using the calibrated normalized relative quantity (CNRQ) method. Four housekeeping genes were assessed using the geNorm algorithm within QBase+ and the two housekeeping genes with lowest mean variability were used as reference genes for sample normalization.

### 2.10 Key resources table

**Table:** 

Reagent or resource	Source	Identifier
**Antibodies**		
Anti-rat endothelial cell antigen 1 (RECA-1), mouse	Abcam	CAT# Ab9774, RRID:AB_296613
Anti-fibronectin, rabbit	Invitrogen	CAT# F3648, RRID:AB_476976
Anti-alpha smooth muscle actin (αSMA), mouse	ThermoFisher	CAT# 14-9760-82, RRID:AB_2572996
Anti-cleaved caspase 3 (CCP3), rabbit	Cell Signalling Technologies	CAT# CST9661, RRID:AB_2341188
Anti-NeuN, mouse	Cell Signaling Technologies	CAT# 94403, RRID:AB_2904530
Anti-GFAP, mouse	Sigma	CAT# G3893, RRID:AB_477010
Anti-collagen IV, rabbit	BioRad	CAT# 2150-1470, RRID:AB_2082660
Anti-IBA1, rabbit	FujiFilm Wako	CAT# 019-19741, RRID:AB_839504
AF488 conjugated anti-rabbit IgG, highly cross-absorbed	ThermoFisher	CAT# A32731, RRID:AB_2633280
AF594 conjugated anti-mouse IgG, highly cross-absorbed	ThermoFisher	CAT# A32742, RRID:AB_2762825
Anti-alpha-actin	Abcam	CAT# Ab179467, RRID:AB_2737344
Anti-IgG HRP conjugated	Abcam	CAT# Ab6721, RRID:AB_955447
**Chemicals, peptides and recombinant proteins**		
Earle’s balanced salt solution	Gibco	CAT# 11540616
HEPES	Fisher Scientific	CAT# 10204932
DMEM cell culture medium	Fisher Scientific	CAT# 11965092
Neurobasal A	Fisher Scientific	CAT# 11540366
B-27	Fisher Scientific	CAT# 11530536
L-glutamine	ThermoFisher	CAT# 25030024
Penicillin-Streptomycin	ThermoFisher	CAT# 15070063
Pentoject, Pentobarbital sodium	Animalcare	CAT# XVD132
Cyanoacrylate glue	RS	CAT# 835-4131
Paraformaldehyde	Merck	CAT# 47608-1L-F
Phosphate buffered saline S (PBS) tablets	Fisher Scientific	CAT# 10209252
Triton X-100	Merck	CAT#X100-1GA
Goat serum	Fisher Scientific	CAT# 11530526
Radioimmunoprecipitation assay (RIPA) buffer	ThermoFisher	CAT# 89900
cOmplete™ Protease Inhibitor Cocktail	Roche	CAT# 11697498001
PhosSTOP Phosphatase Inhibitor	Roche	CAT# 4906845001
Lithium Dodecyl Sulfate (LDS) buffer	ThermoFisher	CAT# NP0007
Sample reducing agent	ThermoFisher	CAT# NP0004
Bis-tris mini protein Gels, 4–12%	ThermoFisher	CAT# NP0322
MOPS SDS running buffer	ThermoFisher	CAT# NP0001
Protran 0.45 NC nitrocellulose Western blotting membranes	Cytiva	CAT# 10600007
NuPAGE Transfer Buffer	ThermoFisher	CAT# NP00061
Skimmed milk powder	VWR	CAT#84615.0500
Tris-buffered saline	Merck	CAT#524750
Tween20	Scientific Laboratory Supplies	CAT#CHE3852
SuperSignal West Pico PLUS Chemiluminescent Substrate	ThermoFisher	CAT# 34580
TRIzol	Invitrogen	CAT# 12044977
Chloroform	ThermoFisher	CAT# J67241.K2
Molecular grade ethanol	Fisher Scientific	CAT#BP2818-212
RNase-Free Water	Qiagen	CAT# 129112
Nuclease-Free Water	Qiagen	CAT# 129117
SYBR Green Universal Master Mix	ThermoFisher	CAT# 43-091-55
**Critical Commercial Assays**		
Bicinchoninic Acid (BCA) Assay Kit	ThermoFisher	CAT# 23225
RNeasy Mini Kit	Qiagen	CAT# 74104
Tetro cDNA Synthesis Kit	Bioline	CAT# BIO65043
**Experimental models: organisms/strains**		
Sprague-Dawley rat	Inotiv	CAT# 002
**Oligonucleotides**		
Primers for quantitative reverse-transcription PCR (RT-qPCR)	Supplementary Table 2	Supplementary Table 2
**Software and Algorithms**		
FIJI	FIJI	https://fiji.sc/
Image processing macro: Percentage coverage of blood vessels	This paper	https://github.com/Dr-ben-hewitt/OBSC-quantification-macros
Image processing macro: Intensity per unit area	This paper	https://github.com/Dr-ben-hewitt/OBSC-quantification-macros
Galaxy	Galaxy	https://usegalaxy.org/
GraphPad Prism 10.3	Graphpad	https://www.graphpad.com/scientific-software/prism
QBase+	CellCarta	https://cellcarta.com/genomic-data-analysis/
Zen 3.1	Zeiss	https://www.zeiss.com/microscopy/en/products/software/zeiss-zen.html
GeneSys / GeneTools	SynGene	https://www.syngene.com/software/

## 3 Results

### 3.1 Health of the OBSCs after 7 days in culture

**FIGURE 2:**
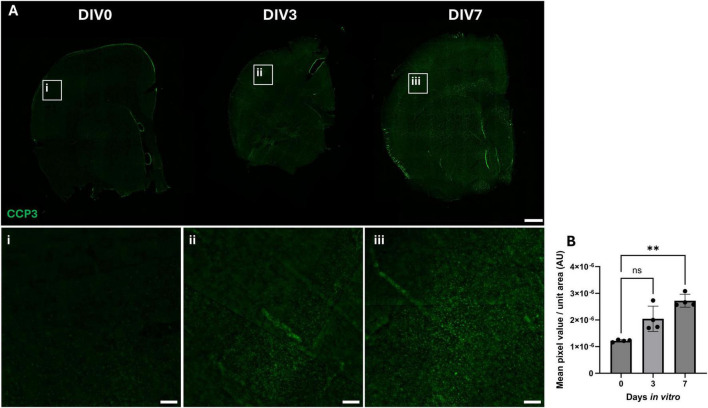
Distribution and intensity of CCP3 throughout control and cultured OBSCs. **(A)** Representative immunofluorescent staining of OBSCs (CCP3, green) at 0 3 and 7 days *in vitro* (scale bar = 1,000 μm). Magnified regions of the cortex of section are shown in insets “i,” “ii,” and “iii” for DIV0, 3 and 7, respectively (scale bar = 100 μm for all). Quantification of mean CCP3-stained pixel intensity per unit area in the cortex was also performed **(B)**. Each image quantification datapoint is mean of six regions of interest from two brain slices per animal (three regions per slice), bars show median ± interquartile range. Statistical testing performed with Brown-Forsythe ANOVA with Dunnett’s T3 multiple comparisons test (all to control) due to non-normal data. ns, not significant; **P < 0.01, *N* = 4 animals, α = 0.05.

Cleaved caspase-3 (CCP3) staining was used as a marker of apoptotic cell death. Overall, there was an increase in CCP3-positive staining from DIV0 to DIV7 in the cortex (Figure 2A) and other areas of the OBSC (Supplementary Figure 3). Quantification of the mean CCP3-positive pixel intensity per unit area revealed a significant increase in CCP3 signal in the cortex (Figure 2B), increasing from 1.21 × 10^–6^ at 0 days to 2.72 × 10^–6^ at 7 days (*P* = 0.0021). A significant increase in CCP3 signal between DIV0 and DIV7 was also noted in the basal ganglia (Supplementary Figure 3).

### 3.2 Alterations in the organotypic cytoarchitecture of the cortex after 7 days in culture

Previous studies suggested OBSCs need to be cultured for 7–14 days prior to use in order to allow the slices to recover from the trauma of vibratome sectioning. Therefore, the cellular composition of OBSCs was assessed at DIV0, DIV3, and DIV7.

**FIGURE 3:**
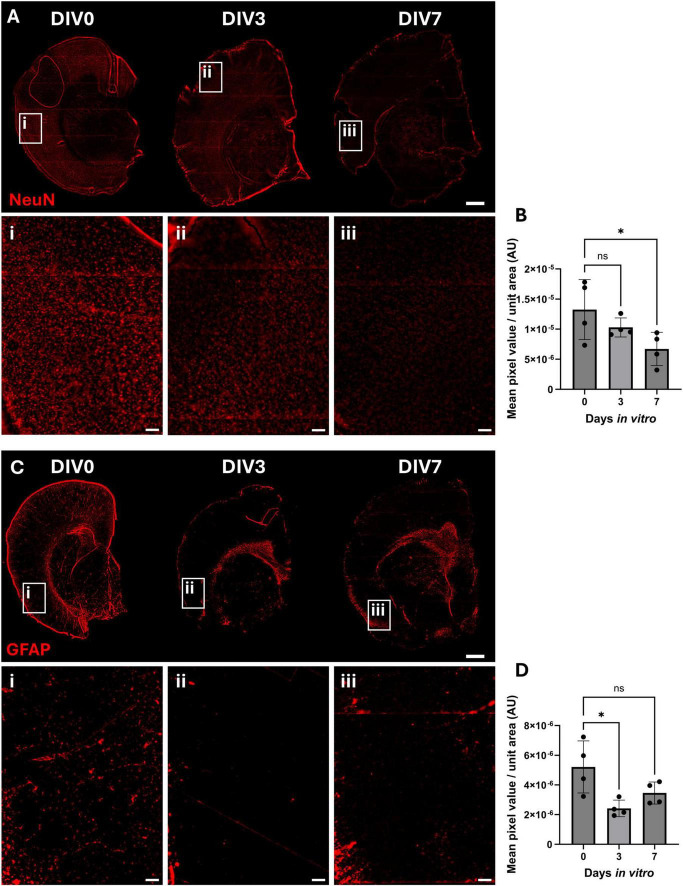
Immunofluorescent staining of neurons and astrocytes in OBSCs after 0, 3, and 7 days *in vitro* (DIV). Representative immunofluorescent staining of **(A)** NeuN (neuronal cell marker) and **(C)** GFAP (astrocyte marker) shown in red (scale bars = 1,000 μm). Magnified regions of the cortex of section are shown in insets “i,” “ii,” and “iii” for DIV0, 3 and 7, respectively (scale bars = 100 μm in insets), in addition to quantification of the mean pixel value per unit area for these regions in insets. **(B)** NeuN quantification in cortex; each datapoint is the mean of five regions of interest per brain slice, bars shown mean ± SD. Statistical testing performed with one-way ANOVA with Dunnett’s multiple comparisons test (all to control). **(D)** GFAP quantification in cortex; each datapoint is the mean of five regions of interest per brain slice, ± SD. Statistical testing performed with one-way ANOVA with Dunnett’s multiple comparisons test (all to control). ns, not significant; *P < 0.05. *N* = 4 animals, α = 0.05.

NeuN, a neuronal cell marker (Figure 3A), demonstrated no significant differences in NeuN staining intensity between DIV0 (mean pixel intensity per unit area of 1.33 × 10^–5^) and DIV3 (1.03 × 10^–5^) (Figure 3B). However, a significant reduction was noted between DIV0 and DIV7 (6.70 × 10^–6^, *P* = 0.0424). In the cortex, glial fibrillary acidic protein (GFAP)-positive astrocytes (Figure 3C) appeared to show a reduction in staining from DIV0 to DIV3, with a particular loss in the glia limitans. This was supported by a significant reduction in quantified GFAP mean pixel value at DIV3 compared to DIV0. Mean pixel intensity per area of GFAP was reduced from 5.21 × 10^–6^ at DIV0 to 2.42 × 10^–6^ at DIV3 (*P* = 0.0132) (Figure 3D). However, no significant difference was seen between DIV0 and DIV7. GFAP-positive astrocytes were maintained within the deeper subcortical white matter (Supplementary Figure 4).

**FIGURE 4:**
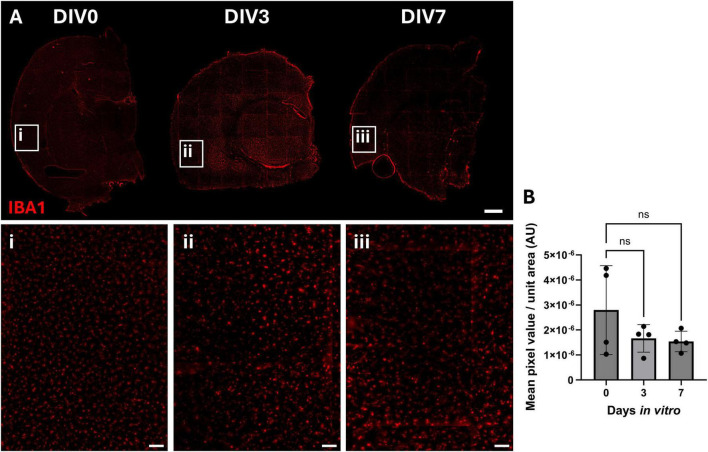
Immunofluorescent staining of microglia in OBSCs after 0, 3, and 7 days in vitro (DIV). **(A)** Representative immunofluorescent staining of IBA1 shown in red (scale bars = 1,000 μm). Magnified regions of the cortex of section are shown in insets “i,” “ii,” and “iii” for DIV0, 3 and 7, respectively, in addition to quantification of the mean pixel value per unit area **(B)** (scale bars = 100 μm in insets). Each datapoint is the mean of five regions of interest per brain slice, ± SD. All statistics were performed with one-way ANOVA with Dunnett’s multiple comparisons test (all to control). ns, not significant. *N* = 4 animals, α = 0.05.

Ionized calcium-binding adapter molecule 1 (IBA1) was used as a microglial cell marker. There was a slight reduction in IBA1-positive cells in the cortex in DIV3 and DIV7 compared to DIV0 (Figure 4A). Image analysis showed no significant reduction after 3 and 7 days of culture (Figure 4B) (*P* > 0.05 for both). In addition, similar to GFAP staining, there did not appear to be any significant changes in IBA1 staining in the subcortical white matter (Supplementary Figure 4).

**FIGURE 5:**
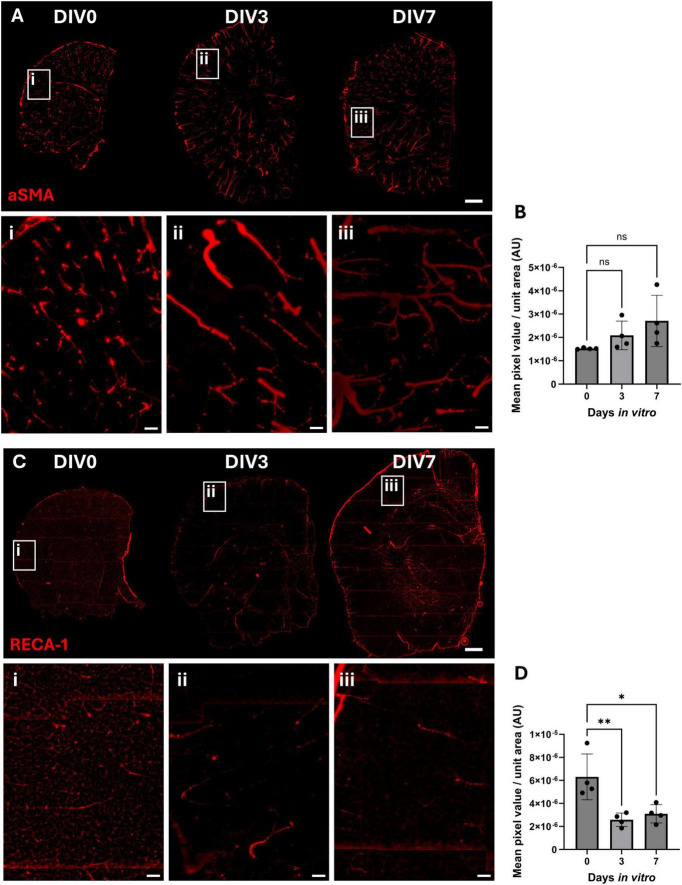
Immunofluorescent staining of smooth muscle cells, pericytes and endothelial cells in OBSCs after 0, 3, and 7 days *in vitro* (DIV). Representative immunofluorescent staining of **(A)** αSMA (alpha smooth muscle actin) and **(C)** rat endothelial cell antigen 1 (RECA-1) shown in red (scale bars = 1,000 μm). RECA-1 representative images are displayed following a 50 pixel rolling ball background subtraction. Magnified regions of the cortex of section are shown in insets “i,” “ii,” and “iii” for DIV0, 3 and 7, respectively (scale bars = 100 μm in insets), in addition to quantification of the mean pixel value per unit area for these regions **(B)** αSMA quantification; each datapoint is the mean of five regions of interest per brain slice, ± SD. Statistical testing performed with one-way ANOVA with Dunnett’s multiple comparisons test (all to control). **(D)** RECA1 quantification; each datapoint is the mean of five regions of interest per brain slice, ± SD range. Statistical testing performed with one-way ANOVA with Dunnett’s multiple comparisons test (all to control). ns, not significant, *P < 0.05, **P < 0.01. *N* = 4 animals, α = 0.05.

Alpha-smooth muscle actin (αSMA) labels vascular smooth muscle cells present within arteries, arterioles and pericytes. The αSMA positive staining pattern showed vessel shaped structures within the brain slice that appeared to be more continuous in DIV3 and DIV7 compared to DIV0 (Figure 5A). However, this was not associated with any significant changes in staining intensity throughout the culture period (Figure 5B).

Finally, rat endothelial cell antigen 1 (RECA-1) staining, which recognizes a cell surface antigen on endothelial cells, showed a reduction in staining of small vessels and capillaries at DIV3 and 7 (Figure 5C). Image analysis (Figure 5D) showed a reduction in mean RECA-1 pixel value per unit area in the cortex, falling from 6.30 × 10^–6^ at DIV0 to 2.58 × 10^–6^ and 3.09 × 10^–6^ at both DIV3 and DIV7 (*P* = 0.0049 and 0.0115, respectively). A similar outcome was shown in the deeper tissues in proximity to the basal ganglia (Supplementary Figure 4).

### 3.3 Blood exposure induced increases in fibronectin and collagen deposits in OBSCs

**FIGURE 6:**
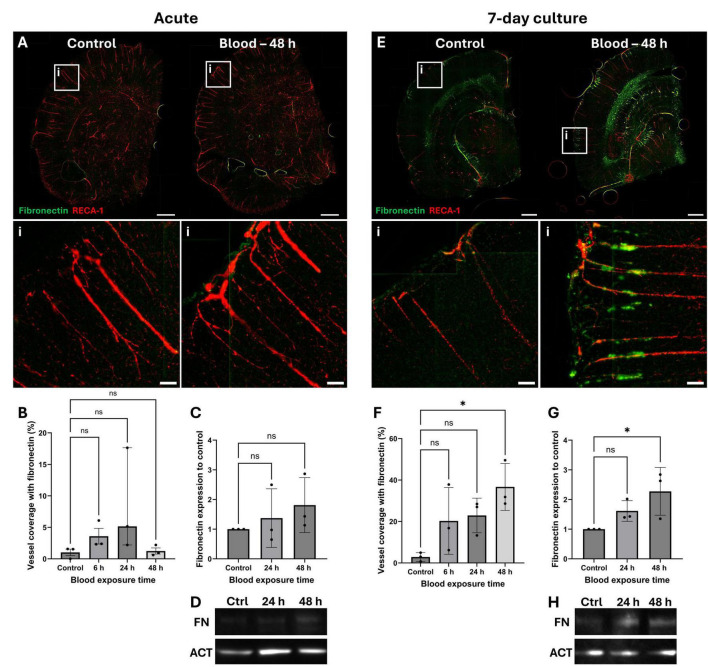
Localization and quantification of fibronectin in blood-exposed OBSCs. OBSCs were generated and cultured either for 0 or 7 days, after which they were exposed either to PBS (control) or the same volume of lysed blood for 48 h. OBSCs were then fixed and stained for RECA1 (red) and fibronectin (green). Representative images of whole hemispheres are shown **(A)** acute, **(E)** 7 day pre-cultured, scale bars = 1,000 μm). Insets show magnified regions of the cortex (i) (scale bars = 100 μm). Quantification of the percentage coverage of fibronectin on RECA1-stained vessels was performed **(B,F)**. Fibronectin protein expression at 24 and 48 h of blood exposure was also assessed by Western blot **(D,H)**, followed by quantification of fibronectin fold-change **(C,G)**. Each image quantification datapoint is the mean of five regions of interest from one brain slice per animal, ± SD, except for **(B)** which is presented as median ± interquartile range. All statistics were performed with one-way ANOVA with Dunnett’s multiple comparisons test (all to control) except for those in **(B)**, which was performed using a Kruskal-Wallis test with multiple comparisons (all to control) due to non-normal data. ns, not significant, *N* = 3 animals, α = 0.05. *P < 0.05.

OBSCs were exposed to lysed blood for 6, 24, or 48 h to mimic hemorrhagic stroke, initiated at either DIV0 (acute) or DIV7 (7-day preculture). Fibronectin staining was shown to be minimal in acute sections (Figure 6A), with image analysis in the cortex and subcortical white matter (Figure 6B and Supplementary Figure 5, respectively), and Western blotting of cortical protein isolates (Figures 6C,D) showing no significant increase in fibronectin staining or expression at any timepoint (*P* > 0.05 for all). However, control OBSCs cultured for 7 days showed visually greater fibronectin staining (Figure 6E) compared to acute sections (Figure 6A). Exposure of these pre-cultured OBSCs to blood resulted in a significant, near 10-fold increase in cortical vessels co-locating with fibronectin, from a mean of 2.97% in control OBSCs to a mean of 28.61% following blood exposure for 48 h (*P* = 0.0123) (Figure 6F). This was further evidenced by a significant increase in fibronectin protein expression as assessed by Western blot, increasing 2.27-fold following blood exposure (*P* = 0.038) (Figures 6G,H). No significant change was noted in fibronectin staining in the subcortical white matter (Supplementary Figure 5).

**FIGURE 7:**
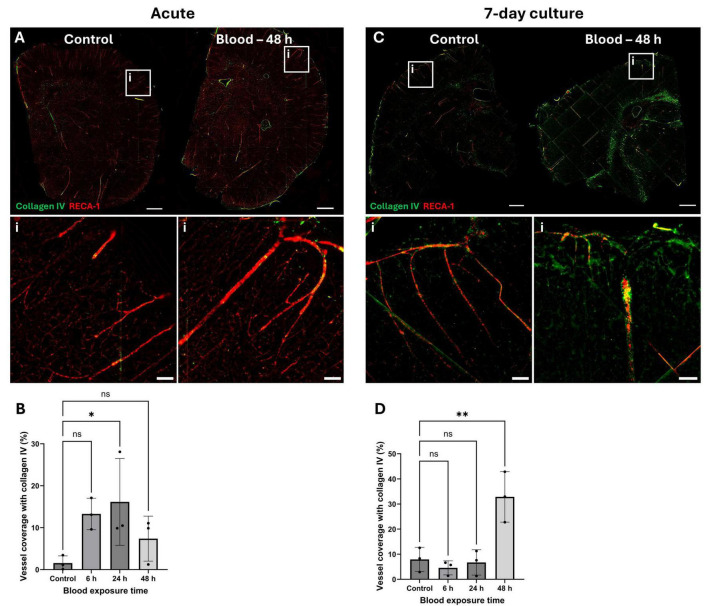
Localization and quantification of collagen IV in blood-exposed OBSCs. OBSCs were generated and cultured either for 0 or 7 days, after which they were exposed either to PBS (control) or the same volume of lysed blood for 48 h. OBSCs were then fixed and stained for RECA1 (red) and collagen IV (green). Representative images of whole hemispheres are shown **(A**, acute, **C**, 7 day pre-cultured, scale bars = 1,000 μm). Insets show magnified regions of the cortex (i) (scale bars = 100 μm). Quantification of the percentage coverage of collagen IV on RECA1-stained vessels was performed **(B,D)**. Each image quantification datapoint is the mean of five regions of interest from one brain slice per animal, ± SD. All statistics were performed with one-way ANOVA with Dunnett’s multiple comparisons test (all to control). ns, not significant, *N* = 3 animals, α = 0.05. *P < 0.05, **P < 0.01.

With collagen IV positive staining, acute OBSCs showing a significant increase in cortical vessel coverage from 1.55% in control samples to 16.15% after 24 h of blood exposure (*P* = 0.0491) (Figure 7B). No significant difference was seen in collagen IV staining in the white matter (*P* > 0.05 for all) (Supplementary Figure 8). In addition, OBSCs treated with blood at DIV7 generated a marked increase in collagen IV signal, increasing from 7.92% cortical vessel coverage by collagen IV in the control to 32.84% after blood exposure (*P* = 0.0032) (Figure 7D).

### 3.4 RNA-sequencing of blood-exposed OBSCs

**FIGURE 8:**
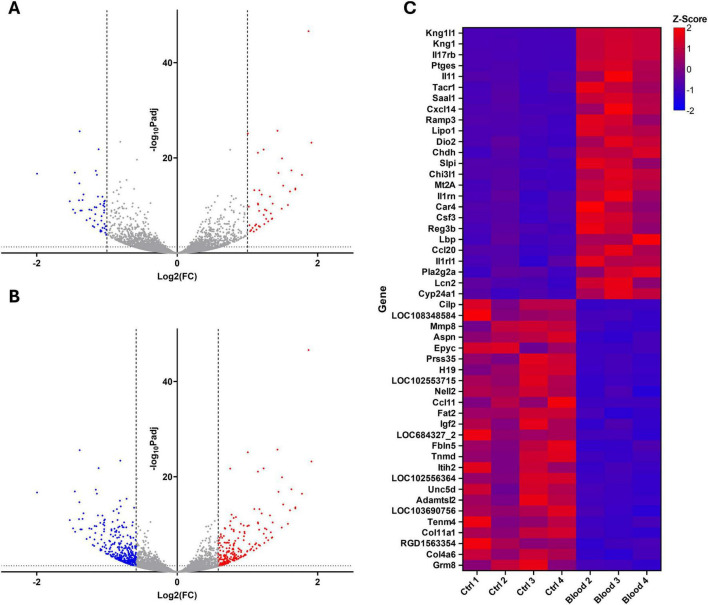
Volcano plots and heatmap of top 25 most differentially expressed genes (DEGs) in control and blood-treated OBSCs. Volcano plots set with a fold change threshold > 2 **(A)** and > 1.5 **(B)** (with fold change thresholds depicted by dotted vertical lines), showing upregulated DEGs (*pAdj* < 0.05) in red and downregulated DEGs in blue. Z-scores for top 25 most up- and down-regulated DEGs (ranked by log_2_ fold change) are visualized via heatmap **(C)**, with upregulated genes shown in red and downregulated genes in blue.

Following sample quality control and principal component analysis (Supplementary Figures 1, 2), one blood-treated sample (B1) was excluded due to sequencing of an insufficient sample quality leading to poor mapping to the reference genome. All subsequent analysis was performed without this sample. Volcano plots were generated for all differentially expressed genes (DEGs) (*pAdj* < 0.05) with expression fold changes (FC) > 2 (Figure 8A) and greater than 1.5 (Figure 8B), with 95 and 604 DEGs over this FC cutoff, respectively. Heatmaps were also generated for each sample across the 25 most upregulated and 25 most downregulated DEGs (Figure 8C).

RT-qPCR validation of the top 6 most upregulated and downregulated genes. Showed a significant decrease in *Aspn*, *Epyc*, *H19*, *Mmp8*, and *Prss35* transcript expression after blood exposure (*P* = 0.0107, 0.0159, 0.0027, 0.026, and 0.0297, respectively. There was no significant reduction in *Cilp* expression, likely due to high inter-sample variability (*P* = 0.2207). Inversely, a significant upregulation of *Il11*, *Ptges*, and *Saal1* was shown (*P* = < 0.0001, 0.0015, and 0.0006, respectively). Increases in *Kng1*, *Il17rb*, and *Tacr* were not significant (*P* = 0.2286, 0.1109, and 0.0667). Overall, 8 of the 12 tested genes showed a significant change in expression (66.7%), with all showing a trend in the same direction as RNA-sequencing data following blood exposure.

**FIGURE 9:**
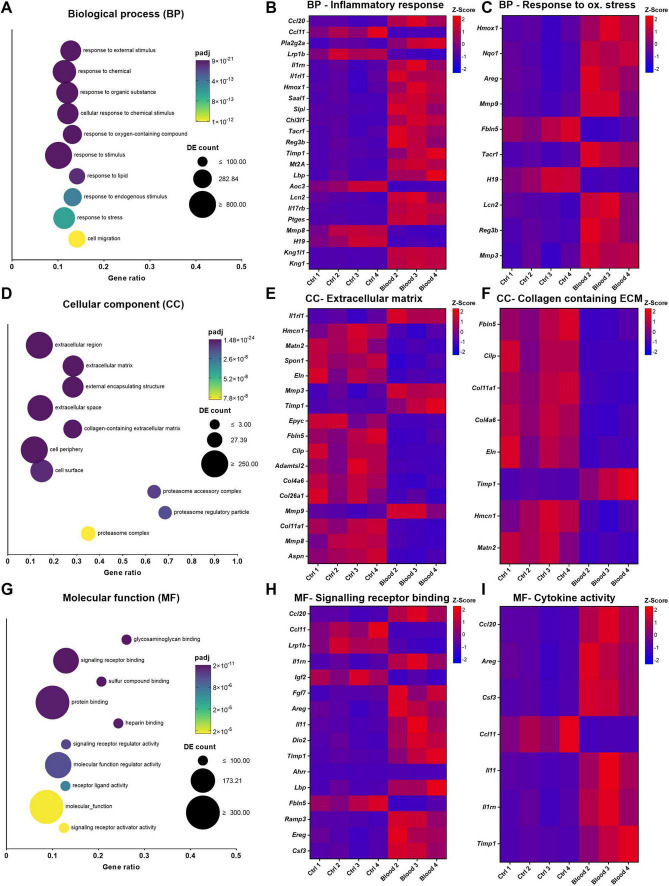
Gene ontology (GO) for control and blood-treated OBSC, with DEGs from selected GO terms within each family. **(A,D,G)** Biological process, cellular component and molecular function GO families depicted with the top 10 most significant overrepresented CC GO terms shown as a bubble plot. The color scale depicts adjusted *p*-value (*padj*) and bubble size represents the number of differentially expressed genes included in the respective category. Gene ratio of 1 = all genes in the GO category are DE, whilst 0 = none. **(B,C,E,F,H,I)** Heatmaps of Z-score of DEGs with FC > 2 or < 0.5 within selected significantly over-represented GO terms “inflammatory response,” “response to oxidative stress,” “extracellular matrix,” “collagen containing extracellular matrix,” “signaling receptor binding,” and “cytokine activity” respectively.

Gene ontology (GO) analysis was then performed across biological process (BP), cellular component (CC) and molecular function (MF) GO families. “Response to external stimulus” and “response to chemical” were the top two most over-represented terms within the BP family (Figure 9D). “Inflammatory response” and “response to oxidative stress” were selected for display (gene ratios 0.141 and 0.149, *padj* 9.64 × 10^–11^ and 1.06 × 10^–7^, respectively), with DEGs from these terms shown in Figures 9E,F, respectively. Blood exposure appeared to cause a marked upregulation of included cytokines and cytokine receptors, including *Ccl20*, *Il1rn*, *Il1r1*, and *Il17rb*. Within the CC family (Figure 9A), “extracellular region” and “extracellular matrix” were the top two most over-represented GO terms (gene ratios 0.137 and 0.285, *padj* 6.45 × 10^–29^ and 8.96 × 10^–27^, respectively). DEGs from extracellular region (Figure 9B) and extracellular matrix terms (Figure 9C) showed downregulation in blood-treated OBSCs including *Col11a1*, *Col4a6*, and *Eln*, downregulation of *Mmp8* and upregulation of *Mmp9*, *Mmp3*, and *Timp1.*

In the MF family, “glycosaminoglycan binding” and “signaling receptor binding” were the topmost over-represented terms (Figure 9G). “Signaling receptor binding” and “cytokine activity” were selected for display (gene ratios 0.129 and 0.168, *padj* 6.55 × 10^–10^ and 5.85 × 10^–5^), with DEGs from these terms shown in Figures 9H,I, respectively. Blood exposure led to an increase in expression of *Il11*, *Csf3*, *Fgf7*, and *Lbp* and a downregulation in *Igf2* and *Fbln5*, as examples.

**FIGURE 10:**
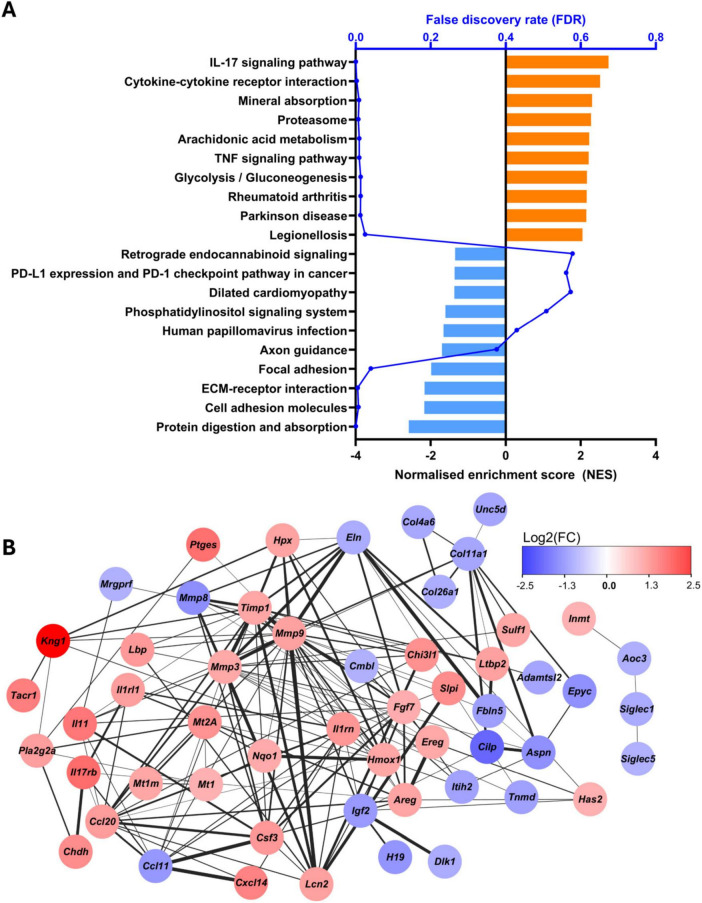
KEGG overrepresentation analysis and protein-protein interaction network. **(A)** KEGG ORA of DEGs, with orange depicting positively enriched terms and light blue showing negatively enriched, plotted by normalized enrichment score (NES) on the bottom X-axis in black. False discovery rate (FDR) is also presented along the top X-axis in blue. **(B)** STRING-enrichment of protein-protein interactions within DEGs (shown as nodes) with non-connected genes excluded. Color depicts Log2(FC) (red = upregulated, blue = downregulated), thickness of edges depicts confidence of protein-protein interaction (thicker = higher confidence).

ORA was performed using the KEGG pathway enrichment method (Figure 10A). Significant positive enrichment was noted in several KEGG terms, including “cytokine-cytokine receptor interaction,” “mineral absorption,” and “proteasome.” Significant negative enrichment was shown for “protein digestion and absorption,” “cell adhesion molecules,” and “ECM-receptor interaction.” Protein-protein interaction, enriched with STRING (Figure 10B), revealed a highly interconnected network of DEGs with 54 nodes (DEGs) and 156 edges (connections), with prominent hub nodes including *Mmp3*, *Mmp9*, *Timp1*, and *Kng1*.

Following this, testing for ECM gene enrichment was performed by comparison of DEGs (both up and downregulated) to the Matrisome ECM gene set (Naba et al., 2012; Table 2). Matrisome genes were significantly over-represented in blood-treated OBSCs, with 2.45-fold enrichment and a Benjamini–Hochberg adjusted *P*-value (pAdj) of 2.61 × 10^–11^ from upregulated DEGs and a 3.41-fold enrichment in downregulated DEGs (pAdj = 5.54 × 10^–33^).

**TABLE 2:** ECM gene enrichment using a curated ECM gene set from the Matrisome project, testing for enrichment amongst all upregulated (Log2FC > 0) and all downregulated (Log2FC < 0) DEGs (pAdj < 0.05).

DEGs	Gene ratio	Fold enrichment	pAdj
Upregulated	64/669	2.450083	2.61E-11
Downregulated	117/879	3.408976	5.54E-33

pAdj = *P*-value after Benjamini–Hochberg multiple-testing correction.

## 4 Discussion

We have described an approach to the generation of adult rat OBSCs and their novel application to the development of an *ex vivo* hemorrhagic stroke model. We demonstrate that blood products have direct effects on ECM regulation in cortical blood vessels at both the transcript and protein level. In addition, we have characterized the distribution and number of several key brain cell types (neurons, endothelial cells, arterial smooth muscle/pericytes, astrocytes and microglia) after 0, 3, and 7 days in culture. All cell types were present across the measured time-course but showed differences in their response to culture conditions.

No significant change was noted in NeuN-positive cell staining after 3 days of culture, but there was a significant loss of neuronal cells at 7 days *in vitro*. In addition, there was a significant reduction in cortical GFAP-positive cells, particularly in the glia limitans, observed between DIV0 and DIV3. A trend toward a reduction was seen between DIV0 and DIV7, though this was no longer significant at this timepoint. This initial loss at DIV3 could be due to either loss of astrocytes or reduction in GFAP expression within astrocytes. The latter seems plausible as the mechanical injury caused by vibratome sectioning has been shown to cause glial cell activation, a hallmark of which is GFAP upregulation (Stence et al., 2001; Pekny and Pekna, 2014). The reduction in GFAP levels observed in our study could therefore represent the return of GFAP to resting levels following an initial slicing-induced spike (Humpel, 2025). Quantification of astrocyte number is required to confirm this, but if correct, this interpretation would support the rationale for culturing brain slices for longer periods of time (Humpel, 2015) before initiating studies in these models. Interestingly, GFAP levels were not reduced everywhere in the brain slice, and GFAP-positive staining was maintained over the time-course within the subcortical white matter. Furthermore, the OBSCs that were cultured for longer periods of time also appeared to show visually greater levels of ECM, particularly in subcortical white matter areas, suggesting that ECM deposition in this area may be related to glial activation. IBA1-positive microglia in the cortex appeared to show a trend toward reduction from DIV0 to DIV3/7, though this was not significant. Previous work has reported a significant reduction in microglial proliferation and an increase in microglial inflammation in the days following slice preparation (Delbridge et al., 2020).

We did observe a significant reduction in RECA-1 staining, particularly associated with capillaries, suggesting a loss of endothelial cells (ECs) from the OBSCs. We suggest that the lack of blood flow, and thus the lack of a pulsatile hemodynamic force and shear stress experienced by the ECs (Li et al., 2005), contributes to this loss. Previous studies have shown that decreased blood flow directly affects EC migration and cytoskeleton reorganization (Vyalov et al., 1996), and causes apoptosis and a reduction in EC number in previously dilated arteries (Sho et al., 2001). Alternatively, our data could be explained by a reduction in the abundance of the RECA-1 epitope (an unknown protein) within endothelial cells which has been suggested by Moser and colleagues (Moser et al., 2003). At the same time, αSMA-positive staining was maintained throughout the 7 days *in vitro*. Furthermore, the staining pattern appeared to be more continuous in DIV3 and DIV7, suggesting that although there wasn’t a change in the amount of αSMA protein, there was potentially more coverage of the associated blood vessels with vascular smooth muscle cells or pericytes. Previous studies show a causative link between reduced blood flow and disordered vascular remodeling (Ward et al., 2001) and increased SMC proliferation (shown initially in hypertensive models) (Ueno et al., 2000).

CCP3 staining increased between DIV0 and DIV7, indicating an increase in apoptotic cell death, which is to be expected due to the acclimatization of slices to culture following the injurious process of vibratome sectioning. This finding does suggest a possible upper limit to the culture period, though longer culture durations were not tested in the present work. Overall, these data suggest the successful maintenance of most tested cell types in OBSCs, with a possible loss of neurons and blood vessel endothelium due to a lack of blood flow through the vasculature. For this reason, future works employing this model (for example, studies of the blood brain barrier or neuronal activity) may choose to employ a shorter pre-culture period, with the caveat of increased astrogliosis and inflammation.

Following this, adult rat OBSCs were cultured for 0 and 7 days and then exposed to lysed rat blood for 6, 24 or 48 h to mimic hemorrhagic stroke. Two key markers of fibrosis, fibronectin (Altrock et al., 2015; Andrews et al., 2018) and collagen IV (Stichel et al., 1999; Klapka et al., 2005), were both significantly elevated along cortical blood vessels (as measured by immunostaining) in the 7-day cultures. 48 h of blood exposure generated the largest response, both on immunofluorescent stains and Western blots. Our results show that these fibrotic deposits are primarily localized to penetrating cortical vessels, where αSMA is most prominent. Interestingly, these deposits were not observed in the 0-day cultures where there was more patchy αSMA staining. This potentially indicating the involvement of SMCs or pericytes in modulating ECM associated with blood vessels. The location of ECM deposits suggest they could contribute to disordered glymphatic flow, reducing the clearance of metabolites (and blood products following hemorrhagic stroke) and exacerbating brain injury. Indeed, perivascular fibrosis has previously been reported in *in vivo* models of ICH (Li et al., 2023a,b) and SAH (Macdonald et al., 1992) and directly linked to reduced clearance of amyloid-β after ischemic stroke (Howe, 2018).

In addition to immunostaining and Western blotting, RNA sequencing was performed on RNA isolated from 7-day precultured OBSCs exposed to blood for 48 h. Blood exposure led to significant over-expression of inflammatory response and cytokine signaling related GO terms, driven by a marked upregulation of inflammation-linked genes, including *Ccl20*, *Il11*, and *Csf3*. *Ccl20* has previously been linked to neuroinflammation following traumatic brain injury (Das et al., 2011) with antagonism shown to reduce this following SAH (Liao et al., 2020). *Il11* concentration in plasma post-ICH has been shown to positively correlate with increased mortality and the development of hydrocephalus (Fang et al., 2005). *Il11* has also been linked to inflammatory immune cell migration in multiple sclerosis (Seyedsadr et al., 2023) and protection against ischemia reperfusion injury in middle cerebral artery occlusion (MCAO) models of ischemic stroke (Zhang et al., 2019). *Csf3* has been suggested to play a role in post-MCAO brain injury by increasing neutrophil toxicity (Garcia-Bonilla et al., 2015). Inflammation-linked KEGG terms “IL-17 signaling pathway” and “TNF signaling pathway” were also shown to be significantly enriched in the positive direction. This large drive towards a pro-inflammatory transcriptome following blood exposure supports studies examining the pro-inflammatory nature of blood and blood products in the subarachnoid space and parenchyma following hemorrhagic stroke (Madangarli et al., 2019; Alsbrook et al., 2023) and suggests that this crucial element is successfully recapitulated in our OBSC-based hemorrhagic stroke model.

Interestingly, the same RNA sequencing data showed a downregulation of ECM-related GO terms and genes (including collagens and elastin), with some upregulation of matrix metalloproteinases (MMPs) and tissue inhibitors of metalloproteinases (TIMPs). KEGG terms “ECM-receptor interaction” and “cell adhesion molecules” were also shown to be significantly enriched in the negative direction, further supporting this. PPI enrichment with DEGs using the STRING interactome revealed an abundance of tightly networked ECM-turnover related genes. Prominent upregulated hubs included *Timp1*, *Mmp9* and *Mmp3*, consistent with proteolytic and ECM regulatory activity, as suggested by GO and KEGG ORA. In contrast, structural ECM components such *Col4a6*, *Col11a1*, and *Col26a1* were shown to be downregulated and were found in more peripheral positions within the PPI network, suggesting a coordinated downregulation of these ECM components. ECM gene over-representation in blood-treated OBSCs was further supported by enrichment using an established ECM gene set (Naba et al., 2012).

Interestingly, immunofluorescent staining and Western blotting of blood-exposed OBSCs suggested a marked increase in ECM deposition along cortical vessels, as previously discussed, suggesting a differential response at the gene and protein level. We propose this increase in protein deposition may not be due to changes in *de novo* protein synthesis, but a manipulation of ECM turnover and deposition triggered by blood-induced changes in the expression of MMPs and TIMPs. Indeed, modified *Mmp9*/*Timp1* expression (as shown in our RNA sequencing data) has been linked to disordered collagen deposition in the skin (Zhou et al., 2021) and lung (Chen et al., 2020). In the brain, a reduction in collagen IV RNA and upregulation of *Mmp9* has previously been reported 48 h after SAH (Solár et al., 2022), a finding reflected in the present work. This discrepancy between gene and protein levels of ECM-related markers may also be caused by other factors, including post-transcriptional regulatory mechanisms. Overall, these findings suggest that our model successfully mimics the ECM dysfunction seen in hemorrhagic stroke, with a likely breakdown of the blood brain barrier and disordered, fibrotic ECM deposition along cortical penetrating vessels.

A limitation of our model is that it simulates global blood exposure instead of localized blood exposure to the cortical tissues or ventricles (as may be seen after SAH or IVH). Further work may be undertaken to identify if this model can recapitulate the differences seen between types of hemorrhagic stroke, i.e., SAH and ICH (Hewitt et al., 2025). Our study also revealed significant reductions in RECA-1 staining of capillary endothelial cells at DIV7, likely driven by the lack of blood flow, suggesting that longer term studies may not be possible. Our study is also limited in its statistical power due to its relatively small sample size, and as such our findings should be interpreted with some caution. Future work may attempt to validate and compare these results in human brain samples from SAH patients with larger cohorts. Single cell RNA sequencing may also be employed to determine which cells drive ECM modulation following blood exposure, as the mechanism underlying this has not been explored in the present work.

## 5 Conclusion

Our findings suggest that our OBSC model can (a) successfully maintain most cell types in adult rat brain slices for at least 7 days and (b) employ lysed blood to recapitulate the disordered ECM deposition (fibrosis) and neuroinflammation which follows hemorrhagic stroke. Future studies may employ this model to further understand the molecular drivers of post-stroke inflammation and fibrosis, or to trial anti-fibrotic agents to reduce ECM dysfunction. Our model offers a platform to advance the translation of pharmacological treatments aimed at reducing post-stroke disability and improving outcomes for stroke survivors, whilst providing a higher throughput and physiologically realistic alternative to *in vivo* animal studies.

## Data Availability

The datasets presented in this study can be found in online repositories. The names of the repository/repositories and accession number(s) can be found at: https://www.ncbi.nlm.nih.gov/, PRJNA1262501.

## Footnotes

1
https://github.com/Dr-ben-hewitt/OBSC-quantification-macros
