# Supplementary material for: Adult organotypic brain slice cultures recapitulate extracellular matrix remodeling in hemorrhagic stroke
Source: Front Cell Neurosci. 2026 Jan 27;19:1722240. doi: 10.3389/fncel.2025.1722240 (PMC12888044; doi:10.3389/fncel.2025.1722240)
Supplement: Supplementary file 1 [file Data_Sheet_1.PDF]

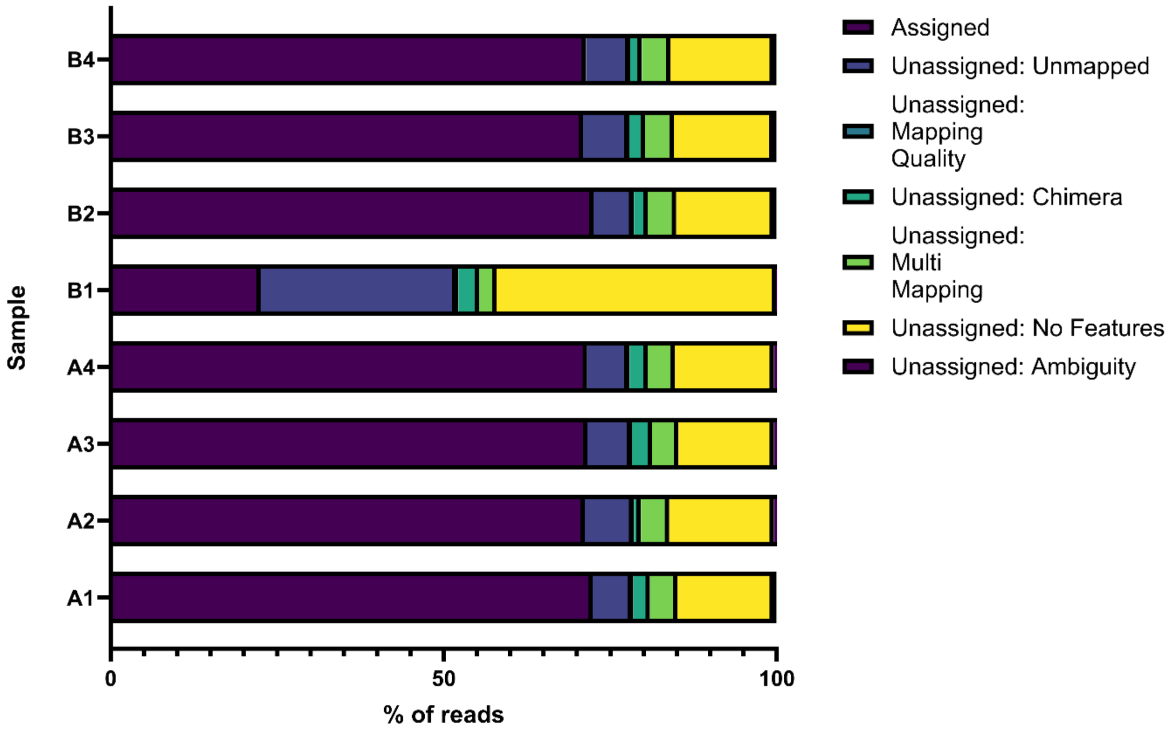

Figure S1 - Mapping of RNA-seq reads to reference genome

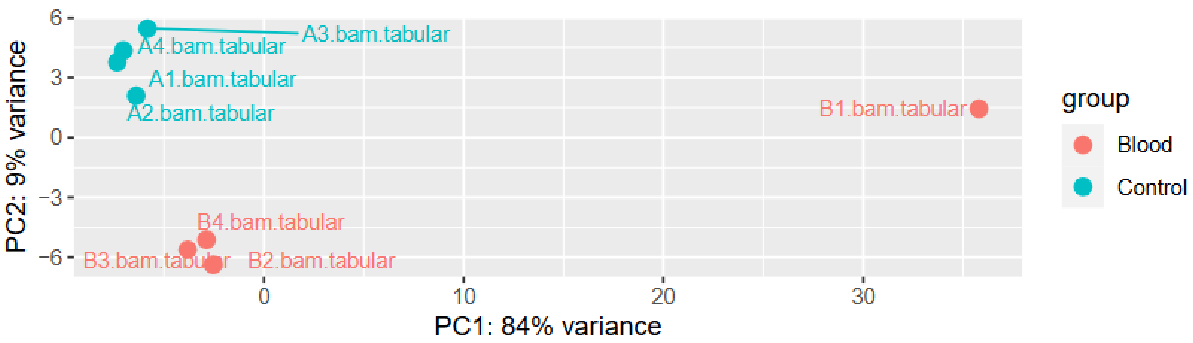

Figure S2 - Principal component analysis (PCA) of control (blue) and blood-exposed (red) OBSC RNA

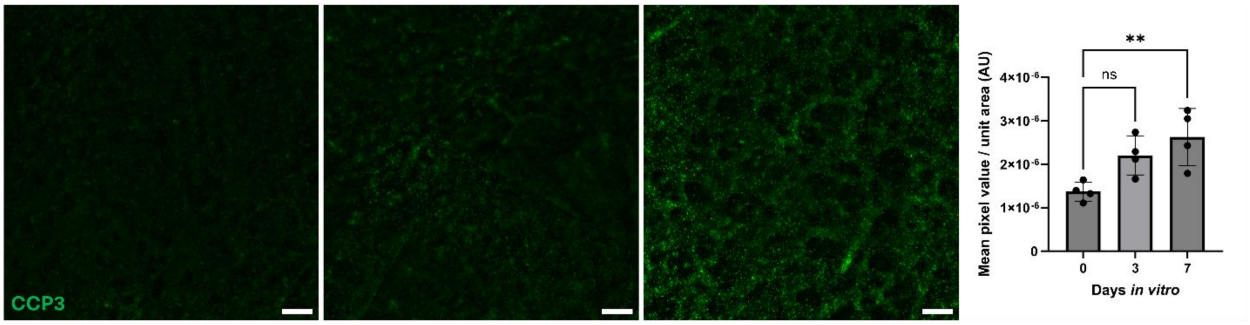

Figure S3 - Distribution and intensity of CCP3 throughout basal ganglia of control and cultured OBSCs. A) Representative immunofluorescent staining of OBSCs (CCP3, green and DAPI, blue) at 0, 3 and 7 days *in vitro* (scale bar = 1000 µm). Magnified regions of the cortex are shown in insert i (scale bar = 100 µm for all). Images processed by background subtraction (50 px radius) and enhancement of window and level for all images equally to improve visibility. Quantification of mean CCP3-stained pixel intensity per unit area in the basal ganglia was also performed (B). Each image quantification datapoint is mean of 6 regions of interest from 2 brain slices per animal (3 regions per slice), ± SD. One-way ANOVA with Dunnett's multiple comparisons test (all to all). ns = not significant, ** $P <$ 0.01. $N = 4$ animals, $\alpha = 0.05$.

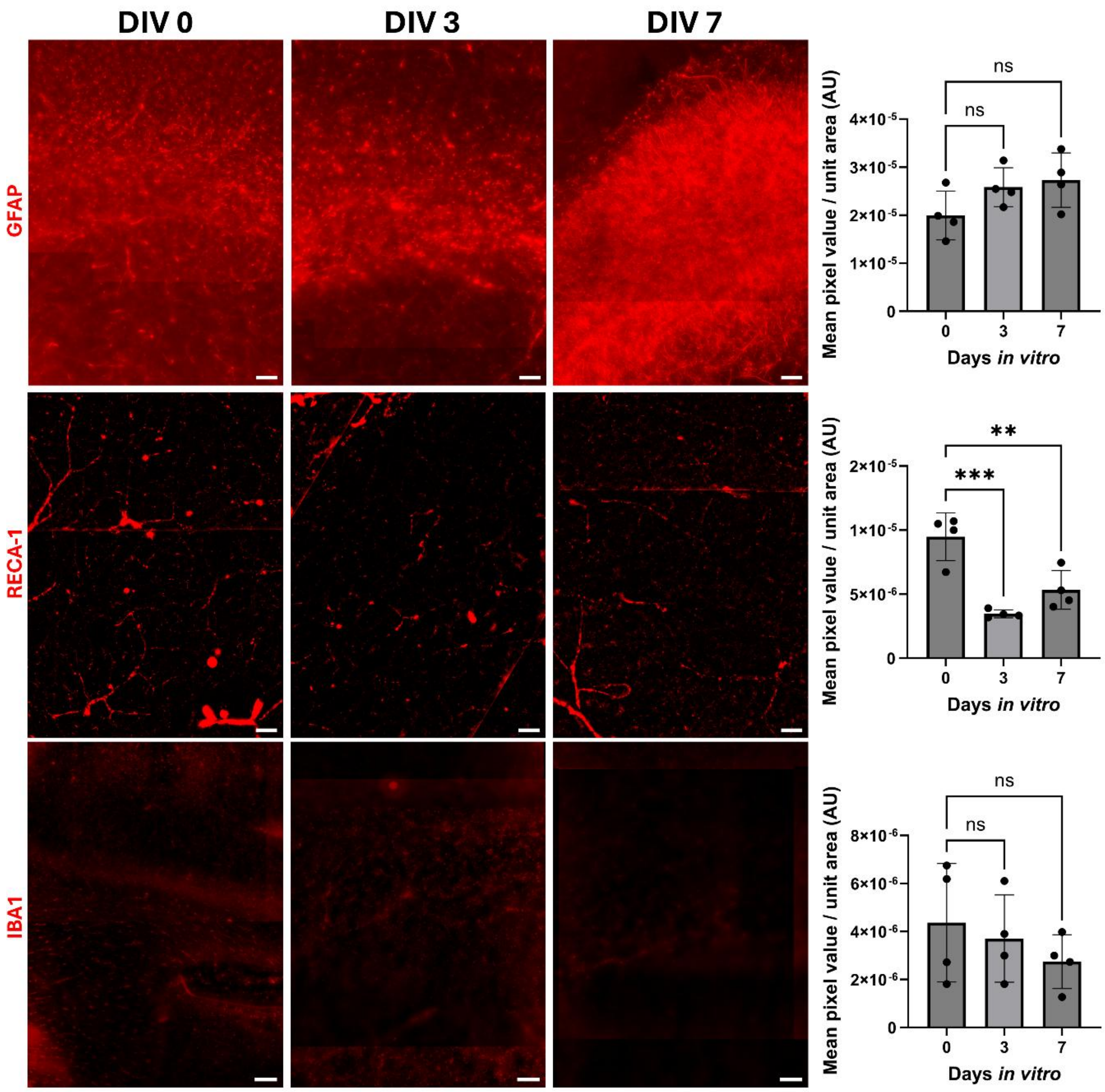

Figure S4 - Immunofluorescent staining of astrocytes, blood vessels and microglia in deeper tissues of OBSCs after 0, 3 and 7 days in vitro (DIV). Regions of interest are sub-cortical white matter for GFAP and IBA1, basal ganglia for RECA-1. Scale bars 100 µm. Quantification of mean stained pixel intensity per unit area was also performed. Each image quantification datapoint is mean of three regions of interest from one brain slice per animal ± SD. One-way ANOVA with Dunnett's multiple comparisons test (all to control). ns = not significant, ** $P < 0.01$, *** $P < 0.001$. $N = 4$ animals, $\alpha = 0.05$.

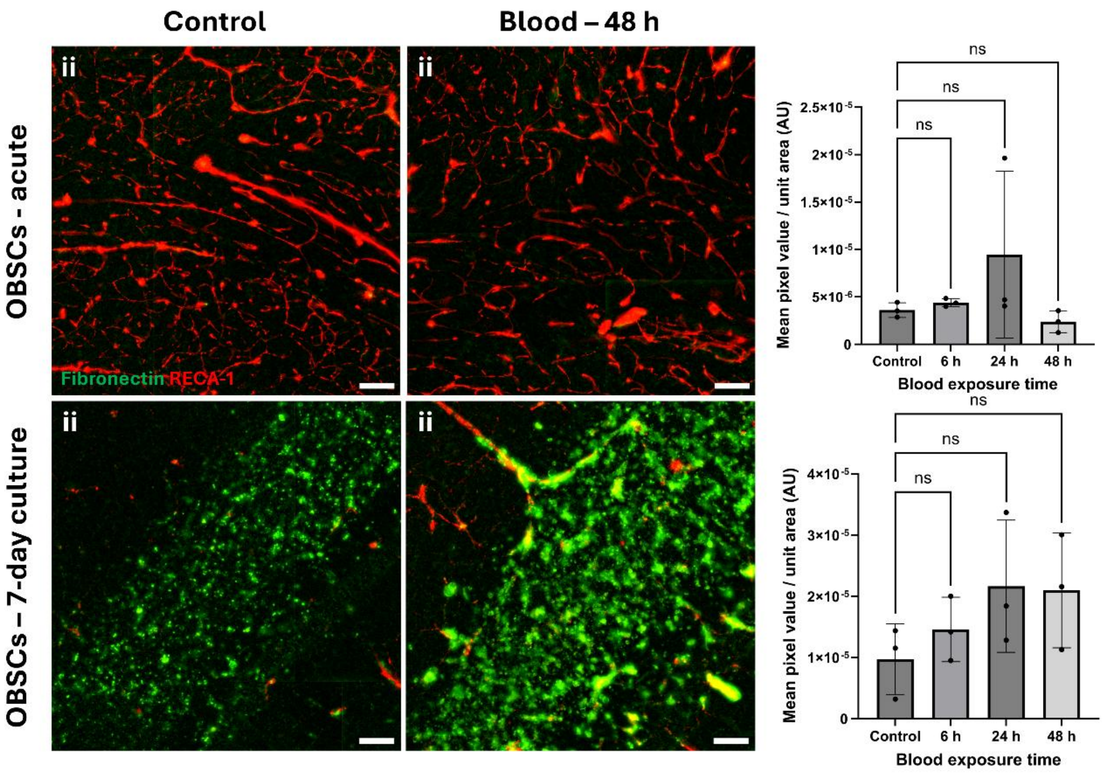

Figure S5 – Localisation and quantification of fibronectin in sub-cortical white matter of blood-exposed OBSCs. OBSCs were generated and cultured either for 0 or 7 days, after which they were exposed either to PBS (control) or the same volume of lysed blood for 48 hours. OBSCs were then fixed and stained for RECA1 (red) and fibronectin (green). Scale bars = 100 µm. Quantification of the percentage coverage of fibronectin on RECA1-stained vessels was performed (B, D). Each image quantification datapoint is mean of three regions of interest from one brain slice per animal, ± SD. All statistics performed with one-way ANOVA with Dunnett's multiple comparisons test (all to control). ns = not significant, N = 3 animals, α = 0.05.

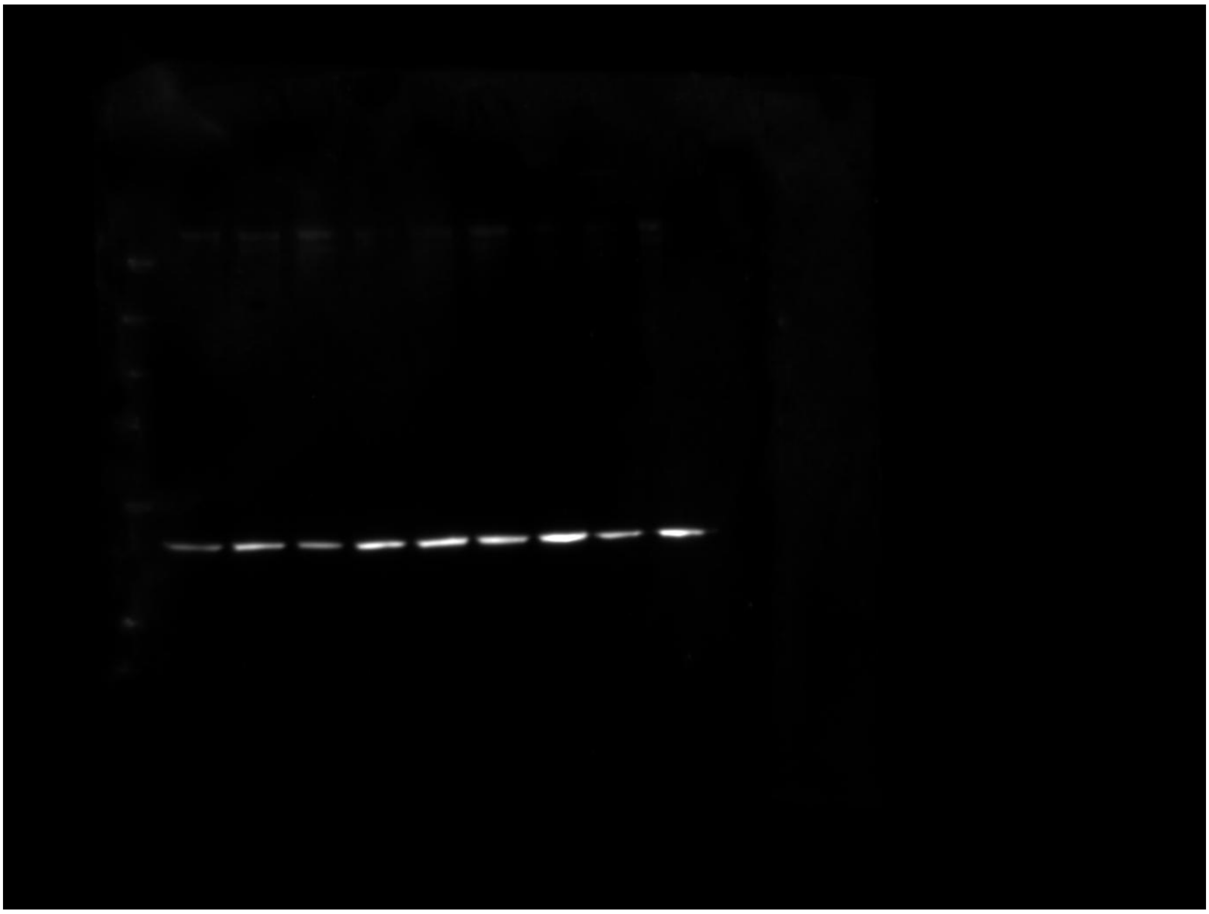

Figure S6 - Unedited, uncropped western blot for acute blood exposed OBSCs. Lanes (L-R)– Ladder, N1 Ctrl, N1 24hrs blood, N1 48hrs blood, N2 ctrl, N2 24 hrs blood, N2 48hrs blood, N3 ctrl, N3 24 hrs blood, N3 48 hrs blood.

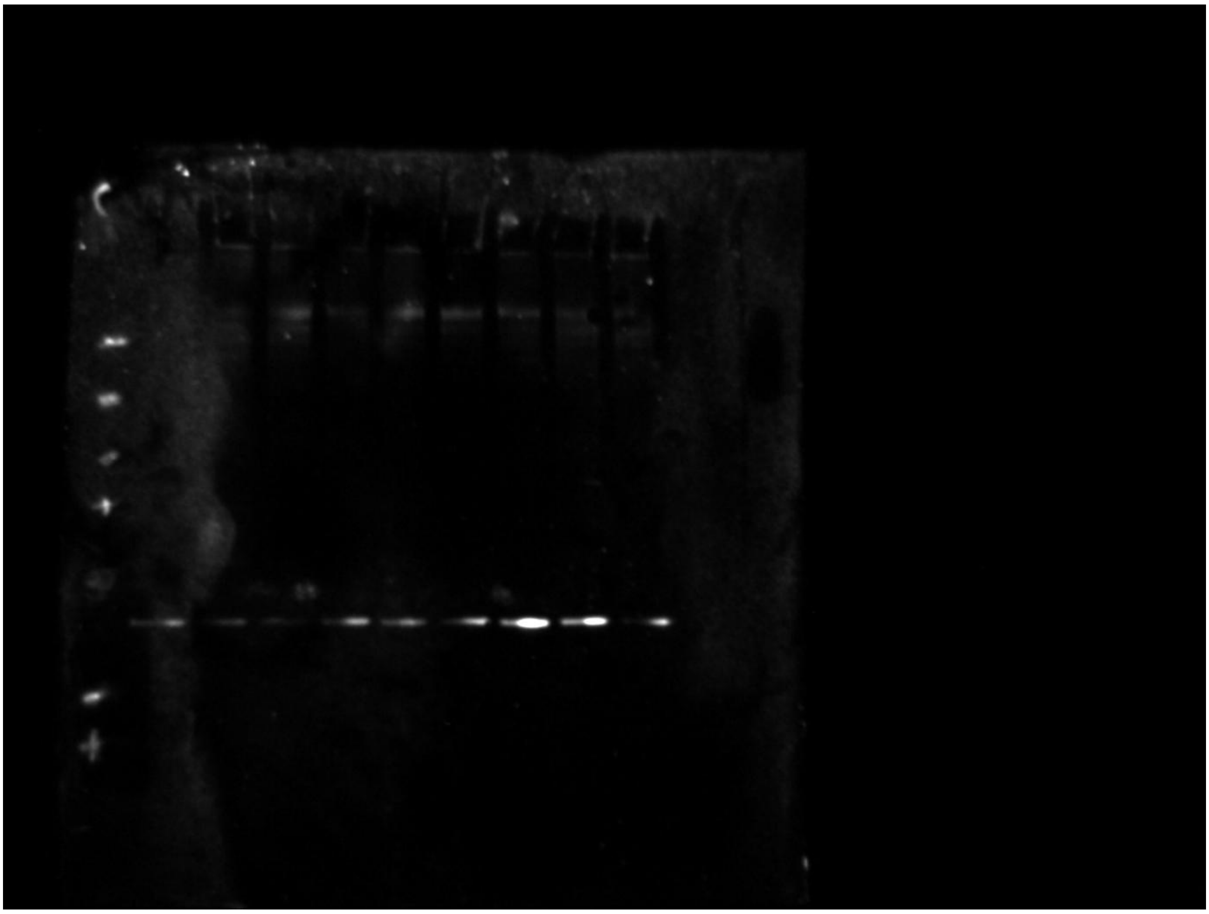

Figure S7 - Unedited, uncropped western blot for 7-day precultured blood exposed OBSCs. Lanes – Ladder, N1 Ctrl, N1 24hrs blood, N1 48hrs blood, N2 ctrl, N2 24 hrs blood, N2 48hrs blood, N3 ctrl, N3 24 hrs blood, N3 48 hrs blood.

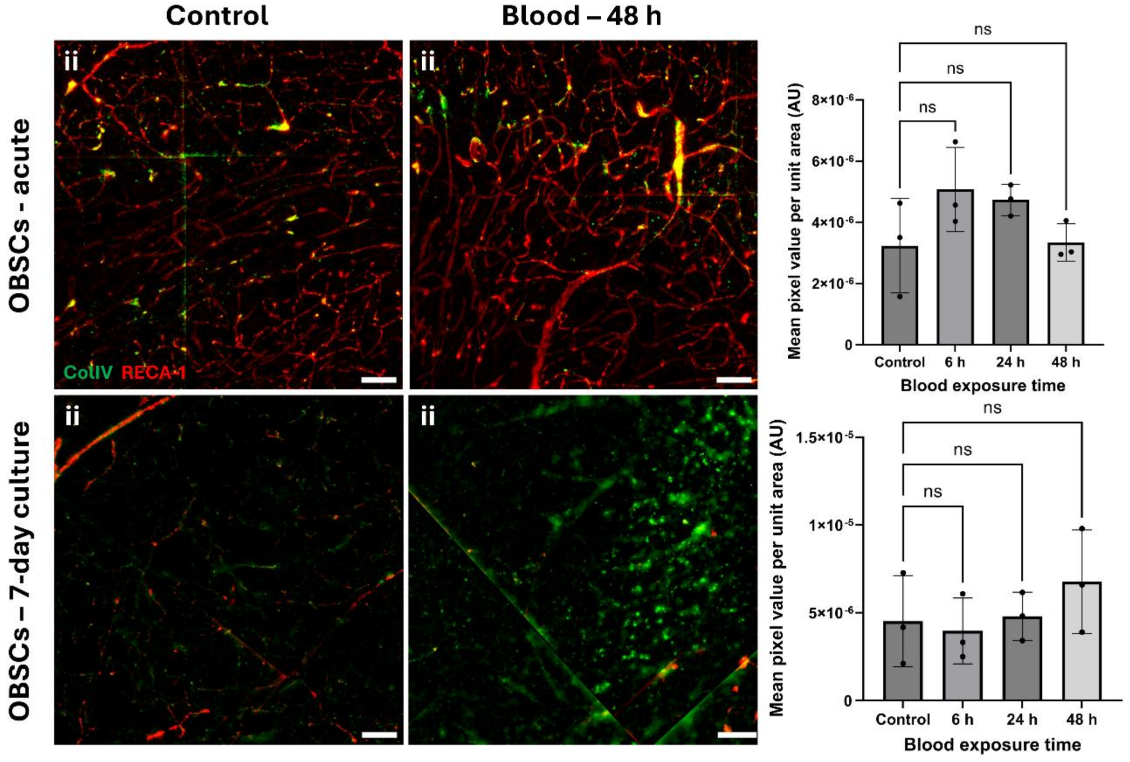

Figure S8 - Localisation and quantification of collagen IV in sub-cortical white matter of blood-exposed OBSCs. OBSCs were generated and cultured either for 0 or 7 days, after which they were exposed either to PBS (control) or the same volume of lysed blood for 48 hours. OBSCs were then fixed and stained for RECA1 (red) and collagen IV (green). Scale bars = 100 µm. Quantification of the percentage coverage of collagen IV on RECA1-stained vessels was performed (B, D). Each image quantification datapoint is mean of three regions of interest from one brain slice per animal, ± SD. All statistics performed with one-way ANOVA with Dunnett's multiple comparisons test (all to control). ns = not significant, N = 3 animals, α = 0.05.

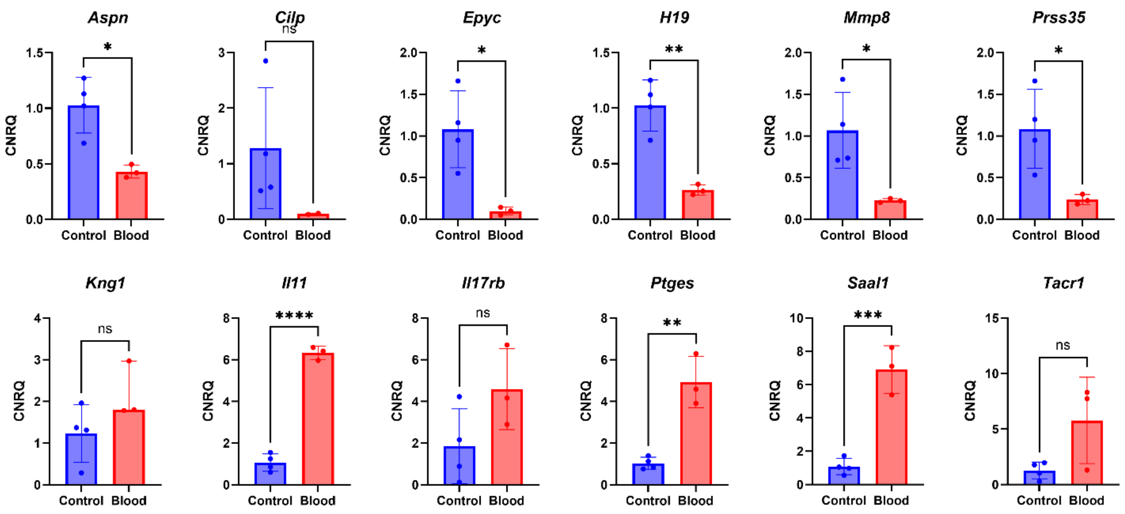

Figure S9 - RT-qPCR validation of RNA-sequencing. Top 6 downregulated (lower expression in blood-exposed samples, top row) and top 6 upregulated (higher expression in

blood-exposed samples, bottom row). CNRQ = calibrated relative normalised quantity. All statistical analyses except for *Kng1* were performed with unpaired t-tests, bars shown mean ±SD. *Kng1* was tested using a Mann-Whitney test due to non-parametric data, shown as median ± interquartile range. ns = not significant, * $P < 0.05$, ** $P < 0.01$, *** $P < 0.001$, **** $P < 0.0001$, $N = 3\text{-}4$, $\alpha = 0.05$.

RT-qPCR validation of RNA-sequencing

Table S1 - RT-qPCR reaction conditions for RNAseq validation.

Reaction step	Temperature (°C)	Ramp rate (°C/s)	Hold time (mm:ss)	Cycles
1 – Preincubation	95	4.4	05:00	1
2 - Amplification	-	-	-	45
2a	90	4.4	00:10	-
2b	60	2.2	00:10	-
2c	72	4.4	00:10	-
3 – Melting Curve	-	-	-	1
3a	95	4.4	00:05	-
3b	65	2.2	01:00	-
3c – Acquisition (5 acquisitions / °C)	65-97	0.11	-	Continuous
4 - Cooling	4	2.2	30:00	1

Table *S*2 - Oligonucleotide primers used in RT-qPCR validation of RNAseq.

Gene symbol	Transcript	Forward (5'-3')	Reverse (5'-3')
KNG1	ENSRNOT00000078131.2	CCAGGGTGCAAGAAGAGAGG	CCCATGCTTATGACCTTGGTG
IL17RB	ENSRNOT00000020823.7	GCATTCATACAGCTGCGTGA	GGGAAGCCGACATAGGAGAA
PTGES	ENSRNOT00055011957.1	GAGGTCTCCAGTACTGCAGG	GATCGTCTCCATGTCGTTGC
IL11	ENSRNOT00000023489.6	CTTCAGACCCTCGTGCAGAT	GCCAAGGTAGGTAGGGAGTC
TACR1	ENSRNOT00000007984.6	GCTGGCCATGAGTTCTACCA	TCCAGCCCCTCATAATCACC
SAAL1	ENSRNOT00000016254.6	CAAATACTTCCATGCTCGGGG	CAGCTCTTGAGTCCTCTGCT
CILP	ENSRNOT00000044887.5	AAGGTCACCCAACTCACTGT	GGTGGCATTCTGGAAGCAAT
MMP8	ENSRNOT00000013936.4	CATAGCAAGCGTGTTCCCAG	AGTGACTCTGCGACTGACAA
ASPN	ENSRNOT00000074041.3	ACGTGTGAGAGAGATCCACT	ATCTTTGGCACTGTTGGACA
EPYC	ENSRNOT00000006450.8	GCAACAACAGACTCGGAAGG	TGGTCCAAGCTGTTATCCGT
PRSS35	ENSRNOT00000034683.5	AGGACACAGCAAGCTTCTCA	TGGTTCCGTTTCAGATCCCA
H19	ENSRNOT00000102917.1	GCTGCTCTCTGGATCCTCTT	GAAGTCCCCGGATTCAAAGG
ACTB	ENSRNOT00000080216.2	TCAGGTCATCACTATCGGCA	AGGTCTTTACGGATGTCAACG
RPL27	ENSRNOT00000028060.7	ACTACAACCACCTCATGCCC	TCCCTGTCTTGTATCGCTCC
RPL13A	ENSRNOT00000093559.2	TGCAAAAGCTTCTGGAGGATG	AGGTGGGAGATGTTGGTCTG
YWHAZ	ENSRNOT00000080676.2	ATGATGGGAGGCAGTGAGTC	TAATTCTCCTCAGCCTGGGA

ImageJ macro language scripts used for image quantification

Also available at - https://github.com/Dr-ben-hewitt/OBSC-quantification-macros

Percentage coverage of blood vessels:

```
// Define the input and output directories

inputDir = getDirectory("Choose the input directory containing CZI files");
```

```
interDir = getDirectory("Choose the intermediate file directory (just needs an empty folder)");
ROIoutputDir = getDirectory("Choose a folder for the ROIs");
outputDir = getDirectory("Choose the output directory for TIFF files");



fileList = getFileList(inputDir);
firstImage = true;
windowMin = 0;
windowMax = 255;
// Loop through each file in the input directory
for (i = 0; i < fileList.length; i++) {
  // Check if the file has a .czi extension
  if (endsWith(fileList[i], ".czi")) {
    // Open the CZI file
    open(inputDir + fileList[i]);
    // Get the title of the current image (used to name the output file)
    title = getTitle();
    origTitle = getTitle();
    noExt = File.nameWithoutExtension;
                if (firstImage) {
    // Wait for the user to adjust the window and level manually
                waitForUser("Open the W/L window, adjust, and click apply. Then click OK.");

    // Get the current window and level values
                getMinAndMax(windowMin, windowMax);

    // Set the firstImage flag to false
                 firstImage = false;
                 }
                else {
    // Apply the saved window and level values to subsequent images
                 setMinAndMax(windowMin, windowMax);
```

```
                        }
            run("Z Project...", "projection=[Max Intensity]");
                        run("8-bit");
                        Stack.setChannel(1);
      run("Green");
      Stack.setChannel(2);
      run("Red");
                        saveAs("OME-TIFF", interDir + "MIP_" + noExt);
                        close(); //added
   }
}
run("Close All");
interFileList = getFileList(interDir);
for (x = 0; x < interFileList.length; x++) {
   // Open the current image
   open(interDir + interFileList[x]);
   // Wait for the user to select ROIs
   Stack.setChannel(2);
   waitForUser("Please select ROIs from image as required, pressing 't' to add them to the manager");
   // Get the number of ROIs
   n = roiManager("count");
   roinum = 1;
   // Loop through all ROIs in the ROI Manager
   for (i = 0; i < n; i++) {
      // Get the current image title and remove extension
      title = getTitle();
      noExt = File.nameWithoutExtension;
      roiManager("Select", i);
      run("Duplicate...", "duplicate");
      saveName = noExt + "_" + "ROI-" + roinum;
      saveAs("OME-TIFF", ROIoutputDir + saveName);
      close();
```

```
      roinum++;
    }
    roiManager("Deselect");
    roiManager("Delete");
}
run("Close All");
////////Split, background subtract, merge ///////////////////////////////////////////////
outputFileList = getFileList(ROIoutputDir);
textDir = getDirectory("Choose the histogram output directory");
textFile = textDir + "output.csv";
File.append("Image Title, Histogram Values\n", textFile);
for (x = 0; x < outputFileList.length; x++) {
                open(ROIoutputDir + outputFileList[x]);
        title = getTitle();
        origtitle = getTitle();
        noExt = File.nameWithoutExtension;
                imageSeq = 0;
    run("Split Channels");
    // PROCESS CH1///////////
    selectWindow("C" + 1 + "-" + title);
    run("Subtract Background...", "rolling=1000");
                run("Auto Threshold", "method=RenyiEntropy white");
    //PROCESS CH2 ////////////////
    selectWindow("C" + 2 + "-" + title);
    run("Subtract Background...", "rolling=100");
    run("Auto Threshold", "method=Triangle white");
    ch1 = "C1-" + title;
    ch2 = "C2-" + title;
    imageCalculator("Average create", ch1, ch2);
    //run("Merge Channels...", "c1=" + ch1 + " c2=" + ch2 + " create");
    merge_title = "Processed_" + noExt;
    rename(merge_title);
```

```
    print("Finished " + merge_title);
    selectWindow(merge_title);
            //// Histogram section
            getHistogram(values, counts, 256);
            line = noExt;
            for (j = 0; j < 256; j++) {
        line = line + counts[j];
        if (j < 255) {
        line = line + ",";
            }
        }
        line = line + "\n";
        File.append(line, textFile);
    selectWindow(merge_title);
            saveAs("OME-TIFF", outputDir + merge_title);
    // Close all images
    run("Close All");
 }

print("Processing complete!");
```

Intensity per unit area:

```
// Define the input and output directories
inputDir = getDirectory("Choose the directory containing the images to be analysed (TIFF format)");
outputDir = getDirectory("Choose a folder for the output");
//
inputFileList = getFileList(inputDir);
textDir = getDirectory("Select the histogram output directory");
textFile = textDir + "GreenQuant.csv";
File.append("Image Title, mean pixel value, area \n", textFile);
for (x = 0; x < inputFileList.length; x++) {
```

```
    // Open the current image
    open(inputDir + inputFileList[x]);
    title = getTitle();
    noExt= File.nameWithoutExtension;
    print(x);
    run("Split Channels");
    selectWindow("C1-" + title);
    run("Subtract Background...", "rolling=50 stack");
    run("Measure");
    meanvalue = getResult("Mean");
    areavalue = getResult("Area");
    saveName = "GREEN-BGSub-50__" + title;
        line = saveName + ","+ meanvalue + "," + areavalue + "\n";
    File.append(line, textFile);
    saveAs("OME-TIFF", outputDir + saveName);
    //
        run("Close All");
}
print ("Quantification done");
```

Staining and clearing of OBSCs

As per manufacturer's instructions. In brief, OBSCs were bleached in 5% (v/v) hydrogen peroxide in methanol:DMSO (80:20%) at 4°C overnight, followed by 30 minutes in antibody penetration buffer (PBS with 0.2% triton-X and 20% DMSO + 0.3M glycine) at room temperature.

Sections were then blocked in PBS with 0.2% triton, 6% goat serum and 10% DMSO for 1 hour at 37°C, followed by primary antibody incubation (diluted in PBS with 0.2% tween20, 10 µg/ml heparin, 3% serum, 5% DMSO) for 90 minutes at room temperature. Washing was performed using PBS with 0.2% Tween20 and 10 µg/ml Heparin. Secondary antibody incubation was then performed for 90 minutes at room temperature, using the same diluent as for primary antibodies. Samples were then washed as previously described, followed by dehydration in graduations of methanol (50, 80, 100%) for 8 minutes each at 4°C. Samples were then mounted in CytoVista tissue clearing reagent following manufacturer's recommendations.

Statistical analysis – normality testing

Figure 2 - Cleaved caspase 3 staining:

Shapiro-Wilk test			
W	0.9528	0.8257	0.7552
P value	0.7334	0.1569	0.0431
Passed normality test (alpha=0.05)?	Yes	Yes	No
P value summary	ns	ns	*

Figure 3 – Cortical NeuN

Shapiro-Wilk test			
W	0.9020	0.8148	0.9530
P value	0.4411	0.1316	0.7347
Passed normality test (alpha=0.05)?	Yes	Yes	Yes
P value summary	ns	ns	ns

Figure 3 – Cortical GFAP

Shapiro-Wilk test			
W	0.9825	0.8894	0.8334
P value	0.9165	0.3804	0.1769
Passed normality test (alpha=0.05)?	Yes	Yes	Yes
P value summary	ns	ns	ns

Figure 4 – Cortical IBA1

Shapiro-Wilk test			
W	0.8299	0.8468	0.9572
P value	0.1675	0.2159	0.7612
Passed normality test (alpha=0.05)?	Yes	Yes	Yes
P value summary	ns	ns	ns

Figure 5 – Cortical aSMA

Shapiro-Wilk test			
W	0.9335	0.8757	0.8968
P value	0.6148	0.3206	0.4153
Passed normality test (alpha=0.05)?	Yes	Yes	Yes
P value summary	ns	ns	ns

Figure 5 – Cortical RECA-1

Shapiro-Wilk test			
W	0.7862	0.9815	0.9829
P value	0.0798	0.9109	0.9186
Passed normality test (alpha=0.05)?	Yes	Yes	Yes
P value summary	ns	ns	ns

Figure 6 – Acute fibronectin image analysis

Shapiro-Wilk test				
W	0.7691	0.7640	0.8868	0.8968
P value	0.0427	0.0311	0.3448	0.3754
Passed normality test (alpha=0.05)?	No	No	Yes	Yes
P value summary	*	*	ns	ns

Figure 6 – Acute fibronectin western blot analysis

Shapiro-Wilk test			
W	Invalid input data	0.8777	0.8751
P value		0.3176	0.3102
Passed normality test (alpha=0.05)?		Yes	Yes
P value summary		ns	ns

Figure 6 – 7-day cultured fibronectin image analysis

Shapiro-Wilk test				
W	0.9986	0.9652	0.8201	0.8619
P value	0.9296	0.6414	0.1635	0.2727

Passed normality test (alpha=0.05)?	Yes	Yes	Yes	Yes
P value summary	ns	ns	ns	ns

Figure 6 – 7-day cultured fibronectin western blot analysis

Shapiro-Wilk test			
W	Invalid input data	0.7897	0.8542
P value		0.0901	0.2519
Passed normality test (alpha=0.05)?		Yes	Yes
P value summary		ns	ns

Figure 7 – Acute Collagen IV image analysis

Shapiro-Wilk test				
W	0.9543	0.9984	0.7751	0.8400
P value	0.5885	0.9236	0.0563	0.2141
Passed normality test (alpha=0.05)?	Yes	Yes	Yes	Yes
P value summary	ns	ns	ns	ns

Figure 7 – 7-day cultured Collagen IV image analysis

Shapiro-Wilk test				
W	0.9952	0.8767	0.9780	0.9999
P value	0.8671	0.3149	0.7155	0.9819
Passed normality test (alpha=0.05)?	Yes	Yes	Yes	Yes
P value summary	ns	ns	ns	ns

Figure S9 – Aspn

Shapiro-Wilk test		
W	0.9465	0.9689
P value	0.6946	0.6614
Passed normality test (alpha=0.05)?	Yes	Yes
P value summary	ns	ns

Figure S9 – Cilp

Shapiro-Wilk test		
W	0.8190	N too small
P value	0.1410	
Passed normality test (alpha=0.05)?	Yes	
P value summary	ns	

Figure S9 – Epyc

Shapiro-Wilk test		
W	0.9935	0.9974
P value	0.9747	0.9020
Passed normality test (alpha=0.05)?	Yes	Yes
P value summary	ns	ns

Figure S9 – H19

Shapiro-Wilk test		
W	0.9533	0.9612
P value	0.7368	0.6215
Passed normality test (alpha=0.05)?	Yes	Yes
P value summary	ns	ns

Figure S9 – Il11

Shapiro-Wilk test		
W	0.9743	0.9363
P value	0.8677	0.5129
Passed normality test (alpha=0.05)?	Yes	Yes
P value summary	ns	ns

Figure S9 - Il17rb

Shapiro-Wilk test		
W	0.9521	0.9632
P value	0.7292	0.6313

Passed normality test (alpha=0.05)?	Yes	Yes
P value summary	ns	ns

Figure S9 – Kng1

Shapiro-Wilk test		
W	0.9285	0.7626
P value	0.5856	0.0280
Passed normality test (alpha=0.05)?	Yes	No
P value summary	ns	*

Figure S9 – Mmp8

Shapiro-Wilk test		
W	0.8707	0.9944
P value	0.3005	0.8564
Passed normality test (alpha=0.05)?	Yes	Yes
P value summary	ns	ns

Figure S9 – Prss35

Shapiro-Wilk test		
W	0.9990	0.9655
P value	0.9972	0.6430
Passed normality test (alpha=0.05)?	Yes	Yes
P value summary	ns	ns

Figure S9 – Ptges

Shapiro-Wilk test		
W	0.9220	0.9417
P value	0.5482	0.5343
Passed normality test (alpha=0.05)?	Yes	Yes
P value summary	ns	ns

Figure S9 – Saal1

Shapiro-Wilk test		
W	0.9450	0.9853
P value	0.6849	0.7682
Passed normality test (alpha=0.05)?	Yes	Yes
P value summary	ns	ns

Figure S9 – Tacr1

Shapiro-Wilk test		
W	0.9282	0.8096
P value	0.5840	0.1376
Passed normality test (alpha=0.05)?	Yes	Yes
P value summary	ns	ns
